# Seven Years of Culture Collection of *Neisseria gonorrhoeae*: Antimicrobial Resistance and Molecular Epidemiology

**DOI:** 10.1089/mdr.2021.0483

**Published:** 2023-03-16

**Authors:** Anna Carannante, Paola Vacca, Stefano Fontana, Ivano Dal Conte, Valeria Ghisetti, Marco Cusini, Grazia Prignano, Caterina Vocale, Anna Maria Barbui, Elena Stroppiana, Marina Busetti, Antonella Mencacci, Marina Rotondi, Maria Antonia De Francesco, Carmen Luciana Bonanno, Patrizia Innocenti, Maria Agnese Latino, Eleonora Riccobono, Federica Poletti, Ines Clotilde Casonato, Graziella Soldato, Luigina Ambrosio, Stefano Boros, Andrea Ciammaruconi, Florigio Lista, Paola Stefanelli

**Affiliations:** ^1^ Department Infectious Diseases, Istituto Superiore di Sanità, Rome, Italy.; ^2^ Department of Prevention, Sexual Health Center, ASL Città di Torino, Turin, Italy.; ^3^ Laboratory of Microbiology and Virology, ASL Città di Torino, Turin, Italy.; ^4^ Dermatology Unit, Fondazione IRCCS Ca’ Granda Ospedale Maggiore Policlinico, Milan, Italy.; ^5^ Molecular Virology, Pathology and Microbiology, IRCCS San Gallicano Dermatological Institute, Rome, Italy.; ^6^ Regional Reference Center for Microbiological Emergencies (CRREM), Unit of Microbiology, St Orsola Malpighi University Hospital, Bologna, Italy.; ^7^ Microbiology and Virology Laboratory, Molinette Hospital, Turin, Italy.; ^8^ Department of Medical Science, Dermatology Clinic, “Città della Salute e della Scienza of Turin,” Turin, Italy.; ^9^ Microbiology Unit, University Hospital of Trieste, Trieste, Italy.; ^10^ Medical Microbiology, Department of Medicine, University of Perugia, Perugia, Italy.; ^11^ Microbiology, Santa Maria della Misericordia Hospital, Perugia, Italy.; ^12^ Clinical and Microbiological Analysis Laboratory, Marilab s.r.l., Rome, Italy.; ^13^ Department of Molecular and Translational Medicine, Institute of Microbiology, University of Brescia-Spedali Civili, Brescia, Italy.; ^14^ Laboratory of Microbiology and Virology, Sandro Pertini Hospital, Rome, Italy.; ^15^ Microbiology and Virology Laboratory, “Comprensorio Sanitario,” Bolzano, Italy.; ^16^ Unit of Bacteriology, Department of “Medicina di Laboratorio," P. O. Sant'Anna, Città della Salute e della Scienza di Torino,” Turin, Italy.; ^17^ Department of Experimental and Clinical Medicine, University of Florence, Florence, Italy.; ^18^ Department Infectious Diseases, Castelli Hospital Verbania, Verbania, Italy.; ^19^ Laboratory of Microbiology, “A. O. Umberto I Mauriziano di Torino,” Turin, Italy.; ^20^ Epidemiology and Public Health Unit, ASL Pescara, Pescara, Italy.; ^21^ Scientific Department, Army Medical Center, Rome, Italy.

**Keywords:** antimicrobial resistance, genogroup, gonorrhea, *N. gonorrhoeae* multiantigen sequence typing (NG-MAST)

## Abstract

The emergence of *Neisseria gonorrhoeae* isolates displaying resistance to antimicrobials, in particular to ceftriaxone monotherapy or ceftriaxone plus azithromycin, represents a global public health concern. This study aimed to analyze the trend of antimicrobial resistance in a 7-year isolate collection retrospective analysis in Italy. Molecular typing on a subsample of gonococci was also included. A total of 1,810 culture-positive gonorrhea cases, collected from 2013 to 2019, were investigated by antimicrobial susceptibility, using gradient diffusion method, and by the *N. gonorrhoeae* multiantigen sequence typing (NG-MAST). The majority of infections occurred among men with urogenital infections and 57.9% of male patients were men who have sex with men. Overall, the cefixime resistance remained stable during the time. An increase of azithromycin resistance was observed until 2018 (26.5%) with a slight decrease in the last year. In 2019, gonococci showing azithromycin minimum inhibitory concentration above the EUCAST epidemiological cutoff value (ECOFF) accounted for 9.9%. Ciprofloxacin resistance and penicillinase-producing *N. gonorrhoeae* (PPNG) percentages increased reaching 79.1% and 18.7% in 2019, respectively. The most common sequence types identified were 5,441, 1,407, 6,360, and 5,624. The predominant genogroup (G) was the 1,407; moreover, a new genogroup G13070 was also detected. A variation in the antimicrobial resistance rates and high genetic variability were observed in this study. The main phenotypic and genotypic characteristics of *N. gonorrhoeae* isolates were described to monitor the spread of drug-resistant gonorrhea.

## Introduction

Antimicrobial resistance is the main issue of *Neisseria gonorrhoeae* and represents a global public health emergency.^1,2^

In 2018, 100,673 cases of gonorrhea were reported by 28 European Union (EU)/European Economic Area (EEA) with the overall notification of 26.4 cases per 100,000 population.^3^ In Italy, 905 confirmed cases were described in 2018, with a rate of 1.5 per 100,000 population.^3^

As described,^3^ gonorrhea cases are likely underestimated and underreported for the majority of EU/EEA countries and, *N. gonorrhoeae* isolates resistant to antimicrobials used previously for treatment (sulfonamides, penicillin, tetracycline, fluoroquinolones) and/or currently in use (third-generation extended-spectrum cephalosporins-ESCs and broad-spectrum macrolides) have been widely spread all over the world.^4–6^

In 2019, the European Gonococcal Antimicrobial Surveillance Program (Euro-GASP)^7,8^ reported a decrease in gonococci resistant to cefixime compared with 2018 (0.9% vs. 1.4%).^8,9^

Ceftriaxone resistance is very rare in Europe and, in 2019, three isolates were described in Norway, Portugal, and Belgium,^8^ instead, gonococci showing azithromycin minimum inhibitory concentration (MIC) above the EUCAST epidemiological cutoff value (ECOFF; MIC >1 mg/L) increased from 7.6% (in 2018) to 10.1% (in 2019) and 15 high-level resistant isolates (HLR, ≥256 mg/L) were reported.^8^

Similarly, in 2019, the ciprofloxacin resistance significantly increased (57.3%) compared with 2018 (50.4%)^8,9^ and the percentage of Penicillin-Producing *Neisseria gonorrhoeae* (PPNG) was 14.9% in 2016, in Europe.^10^

In Italy, the percentages of isolates resistant to cefixime and azithromycin were 17.1% and 23.7%, respectively, in 2009 and decreased to 3.9% and 3% in 2012^11^; no ceftriaxone-resistant isolates were found.^11^ Furthermore, in 2012, 64% of gonococci were resistant to ciprofloxacin and 7% were PPNG.^12^

Many countries report declining *in vitro* susceptibility of azithromycin and ceftriaxone plus azithromycin treatment failures were described, which is a concern because azithromycin and ceftriaxone are the current recommended dual treatment for gonorrhea.^13–15^

Moreover, the Centers for Disease Control and Prevention (CDC)^16^ and the British Association for Sexual Health and HIV (BASHH)^17^ revised the guidelines due to the spread of azithromycin resistance and the emergence of ceftriaxone resistance, among gonococci, worldwide,^14,15,18^ recommending ceftriaxone as monotherapy and the use of azithromycin as a second-line treatment to reduce the selective pressure on gonococcal isolates.^16^

Due to this alarming situation, both the World Health Organization (WHO) and the European Center for Disease Prevention and Control (ECDC)^2,7–9^ have developed action plans to control the spread of antimicrobial resistance in *N. gonorrhoeae* focusing on programs to collect clinical and microbiological data with a special attention to antimicrobial susceptibility pattern.

For this purpose, the surveillance of *N. gonorrhoeae* antimicrobial susceptibility permits to detect emerging and increasing antimicrobial resistance, with particular regard to those suggested for gonorrhea therapy.

*Neisseria gonorrhoeae* Multi-Antigen Sequence Typing (NG-MAST) has been used globally to type gonococcal isolates.^19,20^ As previously described in a pilot study of 2018, the G1407 clone spread in Europe^19^ and globally with a prevalence of 23.3% in the period 2009–2010.^19,20^ This clone is characterized by cefixime and ciprofloxacin resistance^19^ and reduced susceptibility or resistance to azithromycin.^19^ In the 2013 European study,^21^ G1407 was confirmed as the predominant genogroup, however, a decrease in the percentage of ST1407 (7.6% in 2013 vs. 15.6% in 2009–2010) was observed.

The aims of this study are to describe the proportion and trends of *N. gonorrhoeae* resistance to previous and current antimicrobial treatment options, together with the genotypic analysis by NG-MAST. A retrospective evaluation, from 2013 to 2019, on antimicrobial susceptibility and molecular epidemiology of gonococci isolated in Italy is reported in this study.

## Materials and Methods

### *Neisseria gonorrhoeae* isolates and patient data

In this study, from January 2013 to December 2019, 1,810 culture-positive gonorrhea cases were investigated, of which 1,525 (84.2%) viable isolates were available. Primary isolation, identification, and collection of gonococci were completed following standard microbiological procedures by the 18 collaborating laboratories from universities and sexually transmitted infection and dermatology/venereology clinics (11 in the North, 5 in the Center, and 2 in the South of Italy) collaborating with Istituto Superiore di Sanità (ISS). During the study period, 11 collaborating laboratories, out of 18, provided isolates annually; whereas, 4 not for entire period and 3 joined the network since 2018.

Briefly, gonococci, after growth at 37°C in a 5% CO_2_ atmosphere on Thayer–Martin agar plates, were stored at −80°C in brain heart infusion medium (Oxoid, Ltd.) with 20% glycerol. Cultivated isolates were sent bimonthly to ISS.

Unlinked pseudoanonymous data of patients were received and recorded by ISS using Epi Info software (version 3.5.4, 2012).

### Patient characteristics

Taking into account the 7 years, 1,786 patients with one infection episode at a single anatomical site were included. Two episodes of infection were reported for 7 patients and 16 *N. gonorrhoeae* isolates were cultivated and collected in two or three infected anatomical sites for a total of 1,810 culture-positive gonorrhea cases (Supplementary Table S1). The infections were confirmed by culture, microscopy, and/or nucleic acid amplification tests (NAATs). Culture was the most common diagnostic test accounting for 30.6% of confirmed cases. NAAT was used to diagnose 1.6% of cases and microscopy 3.3%. Moreover, it was reported as 21.9% for the use of culture plus microscopy, 17.5% for culture, microscopy plus NAAT, 13.1% for culture plus NAAT (13.1%), and 12.0% for microscopy plus NAAT. Among 1,786 patients, 95.8% were men (median age 34.5 years) and the 57.9% were men who had sex with men (MSM). Women accounted for 4.2% (median age 35.3 years).

The majority of cases (84.6%) occurred among Italians, and during the period, 13 (0.7%) pelvic inflammatory diseases (PID) and 2 (0.1%) disseminated gonococcal infection were reported.

The samples used to diagnose *N. gonorrhoeae* were cervical or urethral discharge; cervical, vaginal, anorectal, urethral, or pharyngeal swabs; blood; urine; seminal fluid; and peritoneal liquid. As shown in Supplementary Table S1, 85.1% were genital infections, 11.5% anorectal, 2.5% pharyngeal, and 0.8% others, including peritonitis as PID complication in a woman.^22^

Finally, the majority of patients reported Italy as a possible country of infection (95.6%).

### Antimicrobial susceptibility testing

All viable isolates were examined for susceptibility to azithromycin, cefixime, ceftriaxone, ciprofloxacin, spectinomycin, and gentamicin by E-test (bioMérieux) and MIC Test Strip (Liofilchem, Italy) in accordance with the manufacturer's instructions, after growth on Thayer–Martin medium (Oxoid, Ltd.), with 1% IsoVitaleX (Oxoid, Ltd.) at 37°C in a 5% CO_2_ atmosphere. Chromogenic reagent nitrocefin (Oxoid, Ltd; Beta-lactamase test, Liofilchem) was used to evaluate the penicillinase production.

After the introduction of the azithromycin ECOFF value, the MIC values were interpreted by referring to both the 2018 and 2021 EUCAST clinical breakpoint criteria (version 8.1, 2018 and version 11, 2021).^23,24^ For testing purposes, the azithromycin ECOFF is 1 mg/L,^23^ and isolates with MIC values >1 mg/L were considered resistant to azithromycin. For gentamicin, the breakpoints were not available. According to the Euro-GASP Reporting Protocol,^7^ for the isolates with cefixime MIC value ≥0.25 mg/L and azithromycin MIC value ≥256 mg/L, the MIC was repeated and the identification was confirmed. The World Health Organization (WHO) *N. gonorrhoeae* G, K, M, O, and P reference strains were used as controls.^25^

### NG-MAST and phylogenetic analysis

Gonococcal DNA was extracted using the QiAmp Mini Kit (Qiagen, Hilden, Germany) from an overnight culture, following the manufacturer's procedure.

NG-MAST was performed on 743 DNAs from viable isolates selected by year (48.7%). All the cefixime and azithromycin-resistant gonococci were included in the analysis. The *porB* and *tbpB* alleles were amplified as previously described^26^ and sequence types (STs) were assigned using the NG-MAST website, following the interpretative procedures.^26^ Closely related STs were defined using the published definition,^19,21^ as well as, the genogroup (G) definition.^19,21^ Multiple sequence alignments were performed using CromasPro version 2.6.6 and the Clustal Omega website.

For each ST, by NG-MAST, excluding the singletons that did not belong to any genogroup, *porB* and *tbpB* sequences were concatenated and imported into the alignment tool. A phylogenetic tree was constructed using MEGA6 software following the maximum likelihood method (Kimura 2-parameter model, gamma distributed).

### Comparative genomic analysis

Whole-genome sequencing (WGS) was performed using the Illumina HiSeq platform, as previously described.^27^ The assemblies were uploaded in the pubMLST database running the BIGSdb genomic platform.^28^

The genetic relationship between a subsample (*n* = 55) of Italian gonococcal genomes, randomly selected, and a panel of *N. gonorrhoeae* genomes (*n* = 521), available from other European countries, were evaluated. Genomes of gonococcal isolates from European countries were selected according to the year of isolation, source of isolation, clinical manifestations, and age of patients available on the PubMLST *Neisseria* database (accessed 12/05/22; See Supplementary Table S2 for the isolate details).

Genome comparison was performed through a gene-by-gene approach using the core genome MLST (cgMLST) scheme, referring to a set of 1,668 loci of the gonococcal genome (*N. gonorrhoeae* cgMLST V.1.0) available on PubMLST.org/neisseria. Incomplete loci were automatically removed from the distance matrix calculation for the neighbor-net graphs. A core genome threshold of 95% were considered for comparison. The resulting distance matrix was visualized as a Neighbor-net in SplitTree4 (version 4.17.1).

### Statistical analysis

*p-value* was calculated by Chi-square for linear trend (Extended Mantel–Haenszel; EpiCalc 2000 statistical calculator) of antimicrobial resistance rate and genogroups. A *p-value* of 0.05 was considered statistically significant. One-way ANOVA statistical analysis was performed by years of data collection to investigate the effect of the year on the MIC values' distribution. The mean was found statistically significant at *p* < 0.0001. Statistical analysis was performed using GraphPad Prism software version 6.0.

## Results

### Antimicrobial susceptibility

**FIG. 1.**
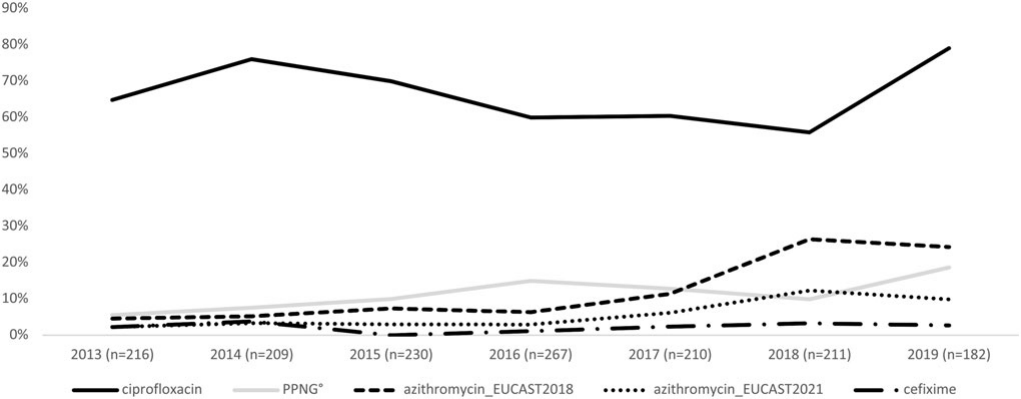
Percentages of resistance of cefixime, azithromycin, ciprofloxacin, and PPNG, from 2013 to 2019. PPNG, penicillinase-producing *Neisseria gonorrhoeae.*

The percentage of resistant *N. gonorrhoeae* by antimicrobial and year on 1,525 viable isolates is shown in Fig. 1.

The proportion of gonococci resistant to cefixime remained quite stable: 2.3% in 2013 versus 2.7% in 2019, (*p* = 0.6454). The lowest cefixime resistance percentage was recorded in 2016 (1.1%) and no resistant gonococci were identified in 2015. During the period, none of the 33 isolates resistant to cefixime showed HLR to azithromycin and a MIC value close to breakpoint of 0.125 mg/L for ceftriaxone.

Referring to 2018 EUCAST guidelines, the azithromycin resistance increased from 4.6% in 2013 to 26.5% in 2018, (*p* < 0.0001) with a slight decrease in 2019 (24.3%). Considering the most recent EUCAST breakpoints published in 2021, the percentage of gonococci showing azithromycin MIC value above the ECOFF of 1 mg/L increased from 2.3% in 2013 to 12.3% in 2018. In 2019, about 10% of the isolates showed a MIC above the ECOFF value for azithromycin. Isolates with MIC values of 0.75 or 1 mg/L were considered susceptible to azithromycin.

Seven azithromycin HLR gonococci (4.7%, MIC ≥256 mg/L), isolated from the urethra of males, were also found. The HLR gonococci were recovered in different years: 2014 (*n* = 1), 2015 (*n* = 2), 2016 (*n* = 2), 2017 (*n* = 1), and 2018 (*n* = 1), respectively.

Ciprofloxacin resistance and PPNG increased from 64.8% and 5.5% in 2013, to 79.1% (*p* = 0.4593), and 18.7% (*p* = 0.0001) in 2019, respectively (Fig. 1).

Ten isolates showed a multidrug-resistant (MDR) pattern for azithromycin, cefixime, and ciprofloxacin. Six gonococci were resistant to azithromycin, ciprofloxacin, and resulted PPNG.

No resistance to ceftriaxone (MIC >0.125 mg/L) or spectinomycin (MIC >64 mg/L) was reported.

The box-plot analysis of ceftriaxone MIC distribution showed that the higher percentage of susceptible gonococci with a MIC value of ≤0.002 mg/L was observed in 2018 (54.5%; median value = 0.002 mg/L), (Supplementary Fig. S1A). The highest MIC value was 0.094 mg/L for one isolate collected in 2014 and the percentage of gonococci with ceftriaxone MIC values close to breakpoint of 0.125 mg/L (MIC = 0.064 or 0–094 mg/L; *n* = 19) decreased from 2.3% in 2013, to 1.6% in 2019. For the spectinomycin MIC distribution, the majority of gonococci showed MIC value of 12 mg/L: 46.9% in 2018 (median value = 12 mg/L) and 39% in 2019 (median value = 12 mg/L), (Supplementary Fig. S1B).

Finally, the box-plot analysis of gentamicin showed the MIC distribution between 4 mg/L and 8 mg/L for the majority of isolates (Supplementary Fig. S1C). In 2017, 29.5% of gonococci showed the MIC value of 4 mg/L (median value = 6 mg/L); in 2018 36.7% showed the MIC value of 6 mg/L (median value = 6 mg/L) and in 2019 38.5% of isolates showed the MIC value of 8 mg/L (median value = 6 mg/L) for this antimicrobial.

For ceftriaxone, spectinomycin, and gentamicin the mean MIC values were found significantly different during the 7-year study period (*p* < 0.0001).

### *Neisseria gonorrhoeae* multiantigen sequence typing

On a subsample of 743 gonococci, 288 STs, of which 182 comprise a single isolate, were identified. One-hundred thirty-seven STs had not been previously described: 76 due to a new *porB* allele, 12 for a new *tbpB* allele, 3 for both of these alleles, and 46 for a new combination of them.

**FIG. 2.**
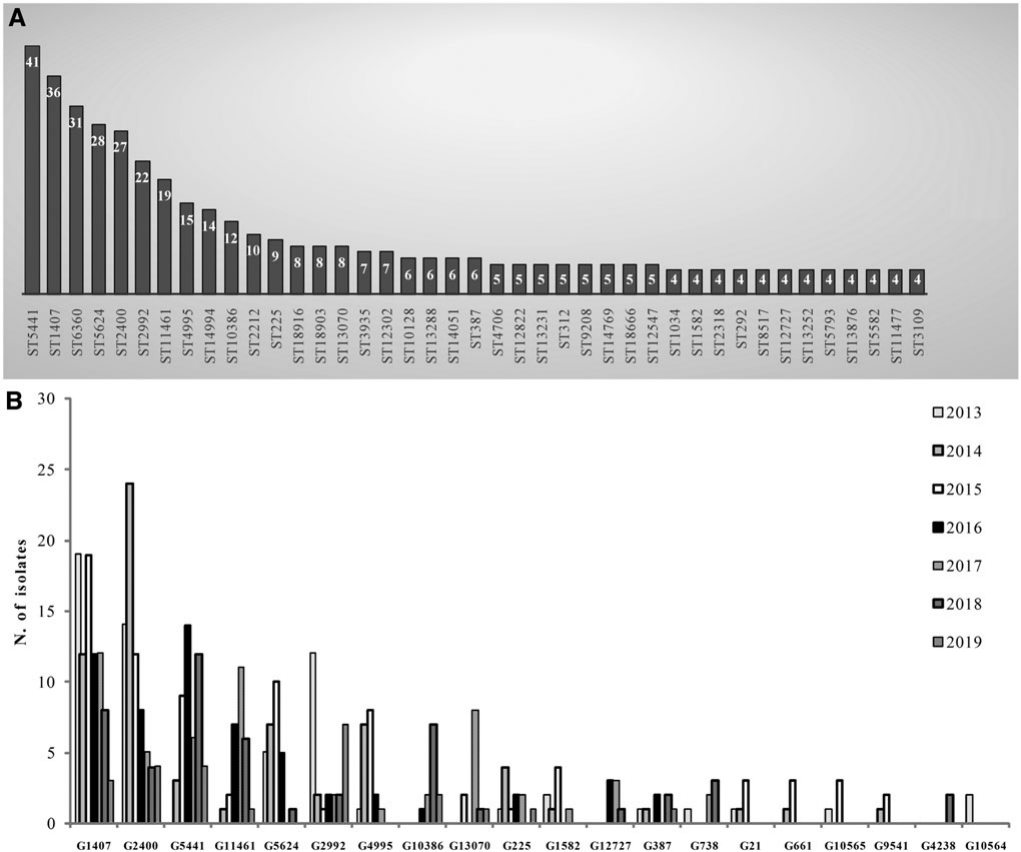
**(A)** Number of STs identified among *N. gonorrhoeae* collected from 2013 to 2019 (only ST comprising at least four gonococci are reported in the Figure). **(B)** Genogroups (G) among *N. gonorrhoeae* isolates by year. ST, sequence type.

The ST5441 was the predominant (*n* = 41, 5.5%) followed by ST1407 (*n* = 36, 4.8%), ST6360 (*n* = 31, 4.2%), ST5624 (*n* = 28, 3.8%), ST2400 (*n* = 27, 3.6%), ST2992 (*n* = 22, 3.0%), ST11461 (*n* = 19, 2.5%), ST4995 (*n* = 15, 2.0%), ST14994 (*n* = 14, 1.9%), ST10386 (*n* = 12, 1.6%), and ST2212 (*n* = 10, 1.3%), (Fig. 2A).

A total of 390 gonococci showed closely related STs and clustered together in 20 different genogroups (G) (Fig. 2B); the STs included in the main genogroups are shown in Table 1.

**Table 1.** Sequence Types Obtained by *Neisseria gonorrhoeae* Multiantigen Sequence Typing Within the 10 Main Genogroups

				(*n* = )						
G1407		G2400	G5441	G11461	G5624	G2992	G4995	G10386	G13070	G225
ST1407 (36)	ST11992 (1)	ST6360 (31)	ST5441 (41)	ST11461 (19)	ST5624 (28)	ST2992 (22)	ST4995 (15)	ST10386 (11)	ST13070 (8)	ST225 (9)
ST2212 (10)	ST12252 (1)	ST2400 (27)	ST13489 (3)	ST14764 (3)		ST5119 (2)	ST10421 (2)	ST16801 (1)	ST13158 (1)	ST3140 (1)
ST4706 (5)	ST12552 (1)	ST10128 (6)	ST16928 (2)	ST15054 (3)		ST5194 (2)	ST11632 (1)		ST16775 (1)	ST735 (1)
ST14695 (3)	ST13073 (1)	ST7437 (2)	ST11746 (1)	ST14696 (2)		ST4684 (1)	ST11658 (1)		ST16797 (1)	
ST10025 (2)	ST13286 (1)	ST11628 (1)	ST18362 (1)	ST16803 (1)		ST5049 (1)			ST18955 (1)	
ST10675 (2)	ST16771 (1)	ST11629 (1)								
ST11630 (2)	ST17010 (1)	ST11714 (1)								
ST1513 (2)	ST3149 (1)	ST11856 (1)								
ST20201 (2)	ST3378 (1)	ST16309 (1)								
ST3431 (2)	ST4843 (1)									
ST5619 (2)	ST4936 (1)									
ST5622 (2)	ST6827 (1)									
ST8826 (2)	ST9829 (1)									

The most frequent were G1407 (represented by 85 isolates), G2400 (represented by 71 isolates), G5441 (represented by 48 isolates), G11461 (represented by 28 isolates), G5624 (represented by 28 isolates), G2992 (represented by 28 isolates), G4995 (represented by 19 isolates), G10386 (represented by 12 isolates), G13070 (represented by 12 isolates), and the G225 (represented by 11 isolates).

G1407 decreased from 22.3% (*n* = 19) in 2013, to 3.5% (*n* = 3) in 2019, (*p* = 0.04469), (Fig. 2B). In particular, within G1407, the predominant ST1407 decreased over the years: from 30.5% in 2013 (*n* = 11) to 5.5% in 2018 (*n* = 2; *p* = 0.0004353), and in 2019 was not found.

In 2014, 33.8% of gonococci belonged to G2400 (*n* = 24, *p* = 0.0003889) and in 2016 the percentage of G5441 accounted for 29.6% of the total (*n* = 14, *p* = 0.0001304) (Fig. 2B).

The percentage of gonococci of the G11461 reached a peak in 2017 (*n* = 11, 39.3%, *p* = 0.0004859) (Fig. 2B); those belonging to G2992 decreased from 2013 (*n* = 12, 42.8%) to 2019 (*n* = 7, 25.0%, *p* = 0.7110), (Fig. 2B).

The 36.8% in 2014 (*n* = 7; *p* = 0.02880), and the 42.1% in 2015 (*n* = 8; *p* = 0.02880), of gonococci belonged to G4995, whereas isolates G5624, including the ST5624, were the most frequent in 2015 (35.7%; *n* = 10, *p* = 0.004568) (Fig. 2B).

G10386, G13070, and G225 reached the higher percentage in 2018 (58.3%; *n* = 7, *p* = 0.00002620), in 2017 (66.6%; *n* = 8, *p* = 0.01463), and in 2014 (36.4%; *n* = 4, *p* = 0.5971) (Fig. 2B), respectively.

All the 33 gonococci resistant to cefixime (isolated from 2013 to 2019) were typed resulting in 22 different STs, of which 13 belonged to G1407 with the prevalent type ST1407 (*n* = 6).

Fourteen gonococci, out of 19 with ceftriaxone MIC values close to breakpoint of 0.125 mg/L, were distributed into 11 different STs, of which 5 belonged to G1407.

From 2013 to 2019, a total of 86 azithromycin-resistant gonococci with MIC value >1 mg/L were identified showing 42 different STs, of which 11 belonged to G2400. ST10128 (*n* = 5) and ST6360 (*n* = 5) were the predominant types.

The seven azithromycin HLR gonococci (MIC ≥256 mg/L) had diverse epidemiological, phenotypic, and molecular characteristics showing low linkages with each other, except for the three isolates with ST3935 (Table 2).

**Table 2.** Details of the Seven *Neisseria gonorrhoeae* Azithromycin High-Level Resistant Isolates (HLR, MIC ≥256 mg/L)

Year	City	MIC (mg/L)						Sequence type
		Azithromycin	Ceftriaxone	Cefixime	Ciprofloxacin	Spectinomycin	Gentamicin	
2014	Rome	≥256	0.008	0.016	≥32	8	12	ST11659
2015	Rome	≥256	0.008	0.016	≥32	6	6	ST11836
2015	Turin	≥256	0.008	0.047	≥32	6	6	ST12822
2016	Milan	≥256	0.006	<0.016	0.016	6	6	ST3935
2016	Turin^a^	≥256	0.008	<0.016	0.023	12	8	ST3935
2017	Milan	≥256	0.006	0.023	0.023	12	8	ST3935
2018	Turin	≥256	0.002	0.016	4	8	12	ST7618

^a^ The isolate was collected in a different collaborating laboratory.

MIC, minimum inhibitory; HLR, high level of resistance.

**FIG. 3.**
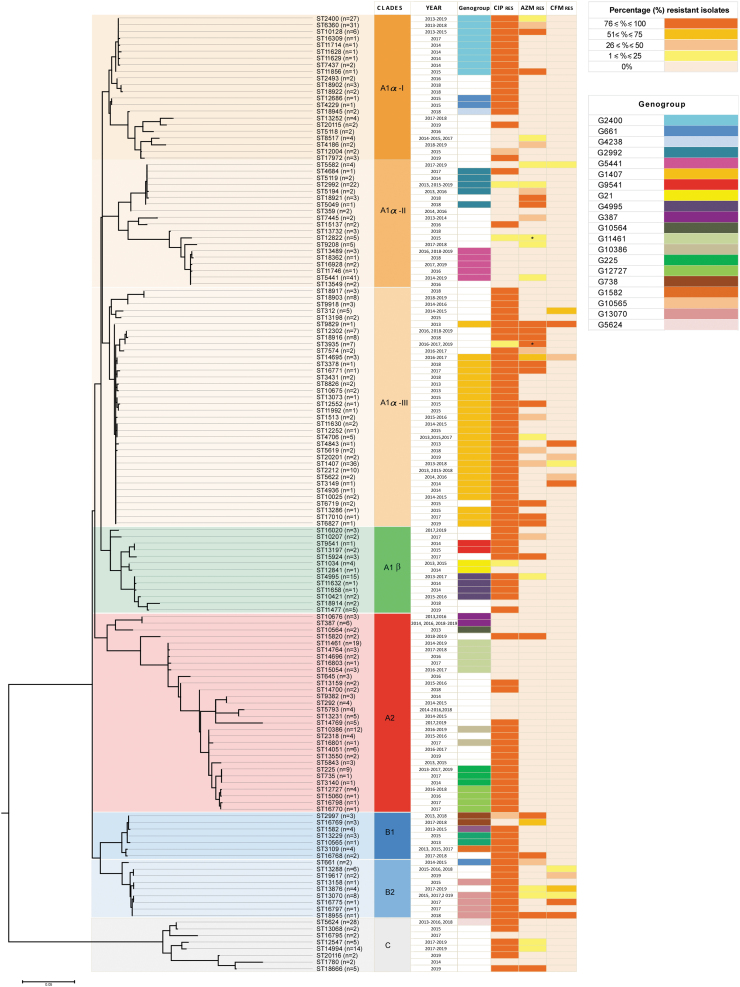
Phylogenetic tree based on the *porB* and *tbpB* sequences (*n* = 144) concatenated of each NG-MAST type, (excluding the singletone STs unless they belong to a genogroup), identified among isolate collected from 2013 to 2019 in Italy. The tree was built using the maximum likelihood method (kimura 2-parameter), and bootstrapped with 100 repetitions. * = presence of HLR for azithromycin. Year, genogroup and ciprofloxacin, azithromycin, and cefixime resistance are indicated. HLR, high level of resistance; NG-MAST, *Neisseria gonorrhoeae* multiantigen sequence typing.

The maximum likelihood phylogenetic analysis grouped the STs into three main clades (A, B, and C), which were divided into subclades, except for the clade C. Figure 3 shows the genetic relationship among *porB* and *tbpB* sequences of 144 STs. All the NG-MAST types found in the analysis were included, except the singletons unless they belonged to a genogroup.

Isolates from clades A1α-I and A1α-III resulted in clones: in these clades were grouped mainly sequences obtained from gonococci ciprofloxacin-resistant G2400 and G1407, respectively.

Sequences of 19 isolates showing ST2400, 6360, 10128, and 11856 of G2400 (52.1%), clustered in the clade A1α-I and were associated with azithromycin resistance pattern (ST2400 4/7 = 14.8%; ST6360 9/31 = 29%; ST10128 5/6 = 83.3%; ST11856 1/1 = 100%). Out of 85 gonococci belonging to G1407, the sequences obtained from gonococci, grouping in clade A1α-III, were associated with azithromycin- (*n* = 24; 28.2%) and cefixime (*n* = 13; 15.3%)-resistant isolates.

Of note, sequences of clade B2 belonged to isolates showing a similar antimicrobial resistance profile of those belonging to G1407. In B2, clustered sequences obtained from gonococci belonging to G13070 were found for the first time in 2015, and from gonococci resistant to ciprofloxacin (*n* = 12; 100%), cefixime (*n* = 3; 25%), and azithromycin (*n* = 2; 16.6%).

### Genomic comparison between Italian and European gonococci

A subsample of 55 genomes were examined by cgMLST for a high-resolution comparison to investigate the genetic relatedness together with a representative panel of genomes from gonococci collected from other European countries (*n* = 521, Supplementary Fig. S2).

The resulting NeighborNet network, reconstructed on the estimated allelic distances, revealed a high genetic variability with a star-like structure, where Italian isolates were intermixed with European gonococci and grouped by genogroup (Supplementary Fig. S2). Of all the European genomes, those from Portugal and Spain were more similar to those collected in Italy (Supplementary Fig. S2).

## Discussion

Antimicrobial resistance in *N. gonorrhoeae* remains a global public health concern, considering that in 2020, the World Health Organization (WHO) estimated an annual incidence of 82 million gonorrhea cases among adults.^29^

To estimate the proportion of resistant *N. gonorrhoeae* isolates and the molecular variability, 1,525 viable gonococci collected in Italy, from 2013 to 2019, were evaluated.

In agreement with previous reports and in EU/EEA countries,^8,9^ most of the gonococcal isolates were collected from the urogenital tract (85.1%) of Italian males (95.8%), the MSM represents the 57.9% and the HIV-positive patients the 14.4%.

As reported in the EU/EEA countries,^9^ the cefixime resistance remained quite stable with a proportion around 2% from 2014 to 2018, reaching the 2.7% in 2019. On the contrary, an increase in azithromycin resistance was detected up to 2018 (from 4.6% in 2013 to 26.5% in 2018). In 2019, referring to the 2018 EUCAST breakpoints for azithromycin, the azithromycin resistance proportion slightly decreased (from 26.5% in 2018 up to 24.3% in 2019). Taking into account the ECOFF value, introduced in the EUCAST guidelines,^22^ in 2019 about 10% of the isolates versus 12.3% observed in 2018, showed a MIC value above the ECOFF value of 1 mg/L.

According to Euro-GASP data published in 2019,^8^ isolates with high level of resistance (HLR) to azithromycin (MIC ≥256 mg/L) were reported also in the country with the last isolate collected in 2018.

Although the European and the International treatment guidelines^13,16^ were updated in 2012, a certain percentage of gonococci resistant to cefixime (2.7%) and azithromycin (9.9%) were identified in Italy in 2019. As reported previously in the country,^11^ despite the combined treatment being in use, the cefixime as monotherapy was prescribed when the guidelines did no longer support its use (after 2013), except when an intramuscular injection was not possible or refused by the patient.^13,16^ Moreover, coinfections with Chlamydia *Chlamydia trachomatis* were likely treated, considering the European treatment guidelines,^13^ with azithromycin as monotherapy.

Unlike other European countries,^8^ no ceftriaxone-resistant isolate was found and the majority of gonococci showed a MIC value of 0.002 mg/L or lower. Nevertheless, during the period of the analysis, 19 gonococci with ceftriaxone MIC values close to the breakpoint of 0.125 mg/L were identified, of which 1 (in 2014) was with MIC value of 0.094 mg/L, and the remaining with MIC value of 0.064 mg/L. The data might suggest a still efficacy of both ceftriaxone and azithromycin to treat gonorrhea.

In Italy, an increase in ciprofloxacin resistance and of PPNG was observed reaching 79.1% and 18.7%, respectively, in 2019. The spread of ciprofloxacin-resistant isolates might likely be the result of its use to treat other infectious diseases.^30^ Finally, less than 1% of gonococci were MDR.

An isolate with high-level resistance to spectinomycin was reported in China, where spectinomycin is used as first-line monotherapy as an alternative to ceftriaxone.^31^

In our country, as well as in Germany and the United Kingdom,^32,33^ no spectinomycin resistance was identified likely due to the limited availability of this antimicrobial, and also capable to select resistant isolates.^5,34^

Gentamicin is still under evaluation for the gonorrhea treatment. A randomized controlled trial reported a noninferiority in the efficacy of gentamicin compared with ceftriaxone in the clearance of *N. gonorrhoeae* in all the anatomic and/or possible other anatomical infected sites, including the urogenital tract.^35^ A breakpoint for gentamicin is not yet established and the gentamicin MIC values, considered in this study, were similar to those reported by other countries.^18,36^

The identification of 288 STs of the gonococci considered in the study, including 182 singleton STs, underlined a high genetic diversity of the gonococci as also previously described (2003–2012).^12^

As also reported,^19,21^ ST1407, ST6360, ST5624, ST2400, ST2992, and ST4995 were widespread in EU/EEA countries, whereas, ST5441 was almost predominant in Italy and previously identified in Spain.^34^

ST13070, also described in Spain^34^ as to be associated with gonococci showing decreased susceptibility or resistance to cefixime and resistance to ciprofloxacin and azithromycin, appeared for the first time in Italy in 2015 reaching a peak in 2017.^37^

According to other reports,^19,21^ and as previously described in our country,^12^ G1407, comprising gonococci resistant to cefixime, ciprofloxacin, and azithromycin,^12,19,21^ remains the predominant genogroup, despite a slowly, but constant decrease over the years (22.3% in 2013 vs. 3.5% in 2019). In fact, a decrease in the percentage of ST1407, one of the main ST of this genogroup (30.5% in 2013 vs. 5.5% in 2018) was observed also in Italy.^21^ G1407 was identified among gonococci with ceftriaxone MIC values close to the breakpoint of 0.125 mg/L. In addition, G2400 was the predominant genogroup among azithromycin-resistant gonococci, with the ST6360 and ST10128 as the most frequent STs. The seven HLR azithromycin isolates showed five different STs of which three of them were ST3935.

Comparison between genomes obtained from gonococci collected in Italy and those from European gonococcal isolates confirmed the high level of genetic diversity of *N. gonorrhoeae* isolates. However, despite this, the majority of circulating gonococci up to 2019 belonged to the distinct genogroups, which are disseminate and spread internationally.

Some limitations have to be mentioned, first of all, the absence of *N. gonorrhoeae* sequence typing for antimicrobial resistance (NG-STAR) and whole-genome sequencing (WGS) performed only on a subsample of gonococci collected in the study period. Harris et al^38^ reported that the genomic analysis will be the method of choice to monitor antimicrobial resistance in *N. gonorrhoeae,* within the surveillance network, due to the comprehensive data available. Second, the study includes only viable culture-positive gonorrhea cases, collected from symptomatic patients, mostly from the urogenital tract.

Overall, the results confirm some important characteristics of the isolates, as previously described,^11,12^ which, however, have to be continuously monitored for circulating gonococci: (i) a stable resistance against cefixime, (ii) a wide-spread resistant isolates to ciprofloxacin and certain proportion of PPNG, (iii) an increase of azithromycin resistance but with a slight decrease in 2019 (the last year of this analysis) and, finally, the lack of ceftriaxone-resistant isolates among the collection analyzed.

The molecular investigation supported the high genetic variability statement for gonococci and the identification of ST13070 and its genogroup, as well as the clear consolidation of the international clone G1407.

Gonococcal AMR surveillance is crucial to monitor antimicrobial susceptibility and to highlight the main circulating clones useful to update, if needed, the treatment guidelines and to reinforce preventive actions.

## Supplementary Material

Supplemental data

Supplemental data

Supplemental data

Supplemental data
