# Supplementary material for: Seven Years of Culture Collection of Neisseria gonorrhoeae: Antimicrobial Resistance and Molecular Epidemiology
Source: Microb Drug Resist. 2023 Mar 16;29(3):85–95. doi: 10.1089/mdr.2021.0483 (PMC10024589; doi:10.1089/mdr.2021.0483)
Supplement: Supplemental data [file Supp_TableS1.docx]

**Table S1.** Patient characteristics

|  | N | (%) |
| --- | --- | --- |
| **Gender** |  |  |
| *Male* | 1634 | 95.8 |
| *Female* | 72 | 4.2 |
| *Unknown* | 80 | |
| **Sexual orientation and gender** |  |  |
| *MSM* | 858 | 57.9 |
| *MSW* | 565 | 38.1 |
| *FSM* | 58 | 3.9 |
| *FSF* | 1 | 0.1 |
| *Unknown* | 304 | |
| **Age (years)** |  |  |
| *<=25* | 364 | 22.2 |
| *>25* | 1273 | 77.8 |
| *Unknown* | 149 | |
| **Citizenship** |  |  |
| *Italian* | 1331 | 84.6 |
| *Western European* | 14 | 0.9 |
| *East European* | 48 | 3.0 |
| *South European* | 49 | 3.1 |
| *Asian* | 17 | 1.1 |
| *Latin American* | 52 | 3.3 |
| *African* | 57 | 3.6 |
| *Others* | 5 | 0.3 |
| *Unknown* | 213 | |
| **Site of sampling*** |  |  |
| *Genital* | 1412 | 85.1 |
| *Anorectal* | 191 | 11.5 |
| *Pharingeal* | 42 | 2.5 |
| *Others* | 14 | 0.8 |
| *Unknown* | 151 | |
| **Previously diagnosed^** |  |  |
| *No* | 1256 | 85.5 |
| *Yes* | 213 | 14.5 |
| *Unknown* | 317 | |
| **Probable country of infection** |  |  |
| *Italy (as reporting country)* | 1280 | 95.6 |
| *Others* | 59 | 4.4 |
| *Unknown* | 447 | |

MSM=men who have sex with men; MSF=men who have sex with females; FSM=females who have sex with men; FSF=females who have sex with females; *= site of sampling was calculated on the total isolates, 1,810; ^considering those diagnosed in the last two months prior to the gonorrhea diagnosis.
