# Supplementary material for: Seven Years of Culture Collection of Neisseria gonorrhoeae: Antimicrobial Resistance and Molecular Epidemiology
Source: Microb Drug Resist. 2023 Mar 16;29(3):85–95. doi: 10.1089/mdr.2021.0483 (PMC10024589; doi:10.1089/mdr.2021.0483)
Supplement: Supplemental data [file Supp_TableS2.docx]

| Table S2. Information on representative gonococcal isolates from Italy and other European countries used for genome comparison | | | | | | | |  |  |
| --- | --- | --- | --- | --- | --- | --- | --- | --- | --- |
| NA, data not available | |  |  |  |  |  |  |  |  |
| **BIGS_ID** | **COUNTRY** | **CONTINENT** | **YEAR** | **AGE_YEAR** | **SEX** | **DISEASE** | **SOURCE** |  |  |
|  |  |  |  |  |  |  |  |  |  |
| 31459 | Slovenia | Europe | 2012 | NA | male | NA | NA |  |  |
| 31517 | Slovenia | Europe | 2012 | NA | male | NA | NA |  |  |
| 31518 | Slovenia | Europe | 2012 | NA | male | NA | NA |  |  |
| 31519 | Slovenia | Europe | 2012 | NA | male | NA | NA |  |  |
| 41197 | UK [England] | Europe | 2012 | NA | NA | uncomplicated gonorrhoea | rectal swab |  |  |
| 41198 | UK [England] | Europe | 2012 | NA | NA | uncomplicated gonorrhoea | urethral swab |  |  |
| 41200 | UK [England] | Europe | 2012 | NA | NA | uncomplicated gonorrhoea | rectal swab |  |  |
| 41210 | UK [England] | Europe | 2012 | NA | NA | uncomplicated gonorrhoea | urethral swab |  |  |
| 46868 | Norway | Europe | 2016 | 61 | female | NA | rectal swab |  |  |
| 46869 | Norway | Europe | 2016 | 50 | female | NA | urethral swab |  |  |
| 46876 | Norway | Europe | 2016 | 49 | male | NA | rectal swab |  |  |
| 46888 | Norway | Europe | 2015 | 21 | male | NA | urethral swab |  |  |
| 46898 | Norway | Europe | 2015 | 24 | male | NA | urethral swab |  |  |
| 46906 | Norway | Europe | 2015 | 23 | male | NA | throat swab |  |  |
| 46914 | Norway | Europe | 2016 | 23 | male | NA | urethral swab |  |  |
| 46925 | Norway | Europe | 2016 | 32 | male | NA | urethral swab |  |  |
| 46930 | Norway | Europe | 2016 | 21 | male | NA | throat swab |  |  |
| 46936 | Norway | Europe | 2016 | 53 | male | NA | urethral swab |  |  |
| 46949 | Norway | Europe | 2016 | 24 | male | NA | urethral swab |  |  |
| 46954 | Norway | Europe | 2016 | 36 | male | NA | urethral swab |  |  |
| 46956 | Norway | Europe | 2016 | 35 | male | NA | throat swab |  |  |
| 47398 | UK [England] | Europe | 2012 | NA | NA | NA | NA |  |  |
| 47417 | UK [England] | Europe | 2012 | NA | NA | NA | NA |  |  |
| 47434 | UK [England] | Europe | 2012 | NA | NA | NA | NA |  |  |
| 47441 | UK [England] | Europe | 2012 | NA | NA | NA | NA |  |  |
| 47532 | UK [England] | Europe | 2012 | NA | NA | NA | NA |  |  |
| 47541 | UK [England] | Europe | 2012 | NA | NA | NA | NA |  |  |
| 47948 | UK [England] | Europe | 2012 | NA | NA | NA | NA |  |  |
| 47953 | UK [England] | Europe | 2012 | NA | NA | NA | NA |  |  |
| 47956 | UK [England] | Europe | 2012 | NA | NA | NA | NA |  |  |
| 47957 | UK [England] | Europe | 2012 | NA | NA | NA | NA |  |  |
| 47958 | UK [England] | Europe | 2012 | NA | NA | NA | NA |  |  |
| 47960 | UK [England] | Europe | 2012 | NA | NA | NA | NA |  |  |
| 47964 | UK [England] | Europe | 2012 | NA | NA | NA | NA |  |  |
| 47967 | UK [England] | Europe | 2012 | NA | NA | NA | NA |  |  |
| 47970 | UK [England] | Europe | 2012 | NA | NA | NA | NA |  |  |
| 47973 | UK [England] | Europe | 2012 | NA | NA | NA | NA |  |  |
| 47977 | UK [England] | Europe | 2012 | NA | NA | NA | NA |  |  |
| 47984 | UK [England] | Europe | 2012 | NA | NA | NA | NA |  |  |
| 47987 | UK [England] | Europe | 2012 | NA | NA | NA | NA |  |  |
| 47991 | UK [England] | Europe | 2012 | NA | NA | NA | NA |  |  |
| 47992 | UK [England] | Europe | 2012 | NA | NA | NA | NA |  |  |
| 47996 | UK [England] | Europe | 2012 | NA | NA | NA | NA |  |  |
| 47998 | UK [England] | Europe | 2012 | NA | NA | NA | NA |  |  |
| 48001 | UK [England] | Europe | 2012 | NA | NA | NA | NA |  |  |
| 48002 | UK [England] | Europe | 2012 | NA | NA | NA | NA |  |  |
| 48007 | UK [England] | Europe | 2012 | NA | NA | NA | NA |  |  |
| 48008 | UK [England] | Europe | 2012 | NA | NA | NA | NA |  |  |
| 48010 | UK [England] | Europe | 2012 | NA | NA | NA | NA |  |  |
| 48019 | UK [England] | Europe | 2012 | NA | NA | NA | NA |  |  |
| 48022 | UK [England] | Europe | 2012 | NA | NA | NA | NA |  |  |
| 48023 | UK [England] | Europe | 2012 | NA | NA | NA | NA |  |  |
| 48025 | UK [England] | Europe | 2012 | NA | NA | NA | NA |  |  |
| 48030 | UK [England] | Europe | 2012 | NA | NA | NA | NA |  |  |
| 48031 | UK [England] | Europe | 2012 | NA | NA | NA | NA |  |  |
| 48034 | UK [England] | Europe | 2012 | NA | NA | NA | NA |  |  |
| 48048 | UK [England] | Europe | 2012 | NA | NA | NA | NA |  |  |
| 48049 | UK [England] | Europe | 2012 | NA | NA | NA | NA |  |  |
| 48051 | UK [England] | Europe | 2012 | NA | NA | NA | NA |  |  |
| 48056 | UK [England] | Europe | 2012 | NA | NA | NA | NA |  |  |
| 48063 | UK [England] | Europe | 2012 | NA | NA | NA | NA |  |  |
| 48065 | UK [England] | Europe | 2012 | NA | NA | NA | NA |  |  |
| 48073 | UK [England] | Europe | 2012 | NA | NA | NA | NA |  |  |
| 48075 | UK [England] | Europe | 2012 | NA | NA | NA | NA |  |  |
| 48078 | UK [England] | Europe | 2012 | NA | NA | NA | NA |  |  |
| 48080 | UK [England] | Europe | 2012 | NA | NA | NA | NA |  |  |
| 48081 | UK [England] | Europe | 2012 | NA | NA | NA | NA |  |  |
| 50489 | Norway | Europe | 2016 | 30 | male | NA | rectal swab |  |  |
| 50491 | Norway | Europe | 2016 | 31 | male | NA | urethral swab |  |  |
| 50513 | Norway | Europe | 2016 | 21 | male | NA | urethral swab |  |  |
| 51103 | Norway | Europe | 2017 | 45 | male | NA | urethral swab |  |  |
| 51105 | Norway | Europe | 2016 | 67 | male | NA | urethral swab |  |  |
| 51117 | Norway | Europe | 2016 | 26 | male | NA | urethral swab |  |  |
| 51686 | Norway | Europe | 2017 | 27 | male | NA | urethral swab |  |  |
| 51695 | Norway | Europe | 2017 | 42 | male | NA | urethral swab |  |  |
| 51714 | Norway | Europe | 2017 | 28 | male | NA | urethral swab |  |  |
| 51718 | Norway | Europe | 2017 | 29 | male | NA | urethral swab |  |  |
| 51719 | Norway | Europe | 2017 | 33 | male | NA | urethral swab |  |  |
| 51724 | Norway | Europe | 2017 | 21 | male | NA | throat swab |  |  |
| 51731 | Norway | Europe | 2017 | 41 | male | NA | urethral swab |  |  |
| 51733 | Norway | Europe | 2017 | 25 | male | NA | urethral swab |  |  |
| 51735 | Norway | Europe | 2017 | 29 | male | NA | urethral swab |  |  |
| 51736 | Norway | Europe | 2017 | 36 | male | NA | urethral swab |  |  |
| 51737 | Norway | Europe | 2017 | 24 | male | NA | urethral swab |  |  |
| 51746 | Norway | Europe | 2017 | 26 | male | NA | urethral swab |  |  |
| 51751 | Norway | Europe | 2017 | 40 | male | NA | urethral swab |  |  |
| 51754 | Norway | Europe | 2017 | 54 | male | NA | urethral swab |  |  |
| 51756 | Norway | Europe | 2017 | 29 | male | NA | urethral swab |  |  |
| 51760 | Norway | Europe | 2017 | 38 | male | NA | urethral swab |  |  |
| 51764 | Norway | Europe | 2017 | 44 | male | NA | urethral swab |  |  |
| 51768 | Norway | Europe | 2017 | 25 | male | NA | urethral swab |  |  |
| 51770 | Norway | Europe | 2017 | 41 | male | NA | urethral swab |  |  |
| 51778 | Norway | Europe | 2017 | 28 | male | NA | urethral swab |  |  |
| 51780 | Norway | Europe | 2017 | 27 | male | NA | urethral swab |  |  |
| 51784 | Norway | Europe | 2017 | 24 | male | NA | urethral swab |  |  |
| 51794 | Norway | Europe | 2017 | 32 | male | NA | urethral swab |  |  |
| 51797 | Norway | Europe | 2017 | 29 | male | NA | urethral swab |  |  |
| 51800 | Norway | Europe | 2017 | 46 | male | NA | urethral swab |  |  |
| 51804 | Norway | Europe | 2017 | 30 | male | NA | urethral swab |  |  |
| 51810 | Norway | Europe | 2017 | 42 | male | NA | urethral swab |  |  |
| 51822 | Norway | Europe | 2017 | 49 | male | NA | urethral swab |  |  |
| 51824 | Norway | Europe | 2017 | 53 | male | NA | urethral swab |  |  |
| 51826 | Norway | Europe | 2017 | 25 | male | NA | urethral swab |  |  |
| 51894 | Norway | Europe | 2017 | 26 | male | NA | urethral swab |  |  |
| 51897 | Norway | Europe | 2017 | 52 | male | NA | urethral swab |  |  |
| 52155 | Norway | Europe | 2017 | 62 | male | NA | urethral swab |  |  |
| 52156 | Norway | Europe | 2017 | 24 | male | NA | urethral swab |  |  |
| 52161 | Norway | Europe | 2017 | 22 | male | NA | urethral swab |  |  |
| 52162 | Norway | Europe | 2017 | 26 | male | NA | urethral swab |  |  |
| 52163 | Norway | Europe | 2017 | 34 | male | NA | urethral swab |  |  |
| 52165 | Norway | Europe | 2017 | 37 | male | NA | urethral swab |  |  |
| 52167 | Norway | Europe | 2017 | 31 | male | NA | urethral swab |  |  |
| 53454 | Norway | Europe | 2017 | 33 | male | NA | urethral swab |  |  |
| 53465 | Norway | Europe | 2017 | 27 | male | NA | urethral swab |  |  |
| 53466 | Norway | Europe | 2017 | 30 | male | NA | urethral swab |  |  |
| 53472 | Norway | Europe | 2017 | 25 | male | NA | throat swab |  |  |
| 53541 | Norway | Europe | 2016 | 27 | male | NA | urethral swab |  |  |
| 53546 | Norway | Europe | 2016 | 22 | male | NA | urethral swab |  |  |
| 53556 | Norway | Europe | 2017 | 47 | male | NA | urethral swab |  |  |
| 53557 | Norway | Europe | 2017 | 46 | male | NA | throat swab |  |  |
| 53565 | Norway | Europe | 2017 | 48 | male | NA | urethral swab |  |  |
| 53566 | Norway | Europe | 2017 | 35 | male | NA | urethral swab |  |  |
| 53569 | Norway | Europe | 2017 | 29 | male | NA | urethral swab |  |  |
| 53571 | Norway | Europe | 2017 | 21 | male | NA | rectal swab |  |  |
| 53586 | Norway | Europe | 2017 | 25 | male | NA | urethral swab |  |  |
| 53588 | Norway | Europe | 2017 | 28 | male | NA | urethral swab |  |  |
| 53591 | Norway | Europe | 2017 | 24 | male | NA | urethral swab |  |  |
| 53601 | Norway | Europe | 2017 | 26 | male | NA | urethral swab |  |  |
| 53609 | Norway | Europe | 2017 | 60 | male | NA | urethral swab |  |  |
| 53612 | Norway | Europe | 2017 | 34 | male | NA | urethral swab |  |  |
| 53619 | Norway | Europe | 2017 | 27 | male | NA | urethral swab |  |  |
| 53626 | Norway | Europe | 2017 | 29 | male | NA | urethral swab |  |  |
| 53631 | Norway | Europe | 2017 | 28 | male | NA | throat swab |  |  |
| 53648 | Norway | Europe | 2017 | 56 | male | NA | urethral swab |  |  |
| 53650 | Norway | Europe | 2017 | 26 | male | NA | urethral swab |  |  |
| 53651 | Norway | Europe | 2017 | 54 | male | NA | urethral swab |  |  |
| 53652 | Norway | Europe | 2017 | 25 | male | NA | urethral swab |  |  |
| 53660 | Norway | Europe | 2017 | 40 | male | NA | throat swab |  |  |
| 53664 | Norway | Europe | 2017 | 22 | male | NA | throat swab |  |  |
| 53667 | Norway | Europe | 2017 | 46 | male | NA | urethral swab |  |  |
| 53669 | Norway | Europe | 2017 | 28 | male | NA | urethral swab |  |  |
| 53673 | Norway | Europe | 2017 | 24 | male | NA | urethral swab |  |  |
| 53675 | Norway | Europe | 2017 | 29 | male | NA | urethral swab |  |  |
| 53677 | Norway | Europe | 2017 | 32 | male | NA | urethral swab |  |  |
| 53679 | Norway | Europe | 2017 | 35 | male | NA | rectal swab |  |  |
| 53680 | Norway | Europe | 2017 | 37 | male | NA | urethral swab |  |  |
| 53799 | Norway | Europe | 2016 | 25 | male | NA | urethral swab |  |  |
| 56131 | Norway | Europe | 2017 | 24 | male | NA | throat swab |  |  |
| 56139 | Norway | Europe | 2017 | 27 | male | NA | urethral swab |  |  |
| 56149 | Norway | Europe | 2017 | 25 | male | NA | urethral swab |  |  |
| 56155 | Norway | Europe | 2017 | 38 | male | NA | urethral swab |  |  |
| 56157 | Norway | Europe | 2017 | 23 | male | NA | throat swab |  |  |
| 56158 | Norway | Europe | 2017 | 34 | male | NA | urethral swab |  |  |
| 56184 | Norway | Europe | 2017 | 24 | male | NA | urethral swab |  |  |
| 56188 | Norway | Europe | 2017 | 25 | male | NA | urethral swab |  |  |
| 56189 | Norway | Europe | 2017 | 30 | male | NA | urethral swab |  |  |
| 56191 | Norway | Europe | 2017 | 69 | male | NA | urethral swab |  |  |
| 56194 | Norway | Europe | 2017 | 41 | male | NA | throat swab |  |  |
| 56195 | Norway | Europe | 2017 | 26 | male | NA | urethral swab |  |  |
| 56196 | Norway | Europe | 2017 | 51 | male | NA | throat swab |  |  |
| 56197 | Norway | Europe | 2017 | 45 | male | NA | urethral swab |  |  |
| 56202 | Norway | Europe | 2017 | 42 | male | NA | rectal swab |  |  |
| 56204 | Norway | Europe | 2017 | 44 | male | NA | rectal swab |  |  |
| 56214 | Norway | Europe | 2017 | 28 | male | NA | urethral swab |  |  |
| 56224 | Norway | Europe | 2017 | 66 | male | NA | throat swab |  |  |
| 56236 | Norway | Europe | 2017 | 54 | male | NA | rectal swab |  |  |
| 56237 | Norway | Europe | 2017 | 39 | male | NA | throat swab |  |  |
| 56239 | Norway | Europe | 2017 | 53 | male | NA | urethral swab |  |  |
| 56241 | Norway | Europe | 2017 | 58 | male | NA | urethral swab |  |  |
| 56246 | Norway | Europe | 2017 | 27 | male | NA | urethral swab |  |  |
| 56247 | Norway | Europe | 2017 | 24 | male | NA | urethral swab |  |  |
| 56262 | Norway | Europe | 2017 | 26 | male | NA | urethral swab |  |  |
| 56271 | Norway | Europe | 2017 | 25 | male | NA | urethral swab |  |  |
| 56272 | Norway | Europe | 2017 | 43 | male | NA | urethral swab |  |  |
| 56273 | Norway | Europe | 2017 | 58 | male | NA | urethral swab |  |  |
| 56277 | Norway | Europe | 2017 | 55 | male | NA | urethral swab |  |  |
| 56279 | Norway | Europe | 2017 | 26 | female | NA | throat swab |  |  |
| 56284 | Norway | Europe | 2017 | 29 | male | NA | urethral swab |  |  |
| 56289 | Norway | Europe | 2017 | 36 | male | NA | urethral swab |  |  |
| 56293 | Norway | Europe | 2017 | 41 | male | NA | urethral swab |  |  |
| 56294 | Norway | Europe | 2017 | 45 | male | NA | rectal swab |  |  |
| 56302 | Norway | Europe | 2017 | 63 | male | NA | rectal swab |  |  |
| 56306 | Norway | Europe | 2017 | 34 | male | NA | rectal swab |  |  |
| 56773 | Spain | Europe | 2016 | 44 | male | uncomplicated gonorrhoea | rectal swab |  |  |
| 56776 | Spain | Europe | 2016 | 35 | male | uncomplicated gonorrhoea | urethral swab |  |  |
| 56777 | Spain | Europe | 2016 | 35 | male | uncomplicated gonorrhoea | throat swab |  |  |
| 56778 | Spain | Europe | 2016 | 43 | female | uncomplicated gonorrhoea | NA |  |  |
| 56779 | Spain | Europe | 2016 | 43 | female | uncomplicated gonorrhoea | throat swab |  |  |
| 56780 | Spain | Europe | 2016 | 41 | male | uncomplicated gonorrhoea | throat swab |  |  |
| 56781 | Spain | Europe | 2016 | 41 | male | uncomplicated gonorrhoea | rectal swab |  |  |
| 56782 | Spain | Europe | 2016 | 41 | male | uncomplicated gonorrhoea | urethral swab |  |  |
| 56783 | Spain | Europe | 2016 | 41 | male | uncomplicated gonorrhoea | throat swab |  |  |
| 56784 | Spain | Europe | 2016 | 23 | male | uncomplicated gonorrhoea | rectal swab |  |  |
| 56785 | Spain | Europe | 2016 | 23 | male | uncomplicated gonorrhoea | throat swab |  |  |
| 56786 | Spain | Europe | 2016 | 46 | female | uncomplicated gonorrhoea | NA |  |  |
| 56787 | Spain | Europe | 2016 | 46 | female | uncomplicated gonorrhoea | throat swab |  |  |
| 57092 | Spain | Europe | 2016 | 26 | male | uncomplicated gonorrhoea | rectal swab |  |  |
| 57093 | Spain | Europe | 2016 | 23 | female | uncomplicated gonorrhoea | NA |  |  |
| 57094 | Spain | Europe | 2016 | 43 | male | uncomplicated gonorrhoea | urethral swab |  |  |
| 57095 | Spain | Europe | 2016 | 29 | female | uncomplicated gonorrhoea | NA |  |  |
| 57096 | Spain | Europe | 2016 | 31 | male | uncomplicated gonorrhoea | rectal swab |  |  |
| 57097 | Spain | Europe | 2016 | 30 | male | uncomplicated gonorrhoea | urethral swab |  |  |
| 57098 | Spain | Europe | 2016 | 44 | male | uncomplicated gonorrhoea | urethral swab |  |  |
| 57099 | Spain | Europe | 2016 | 25 | female | uncomplicated gonorrhoea | throat swab |  |  |
| 57100 | Spain | Europe | 2016 | 35 | male | uncomplicated gonorrhoea | urethral swab |  |  |
| 57102 | Spain | Europe | 2016 | 36 | male | uncomplicated gonorrhoea | urethral swab |  |  |
| 57103 | Spain | Europe | 2016 | 29 | male | uncomplicated gonorrhoea | rectal swab |  |  |
| 57104 | Spain | Europe | 2016 | 48 | male | uncomplicated gonorrhoea | throat swab |  |  |
| 57105 | Spain | Europe | 2016 | 30 | male | uncomplicated gonorrhoea | urethral swab |  |  |
| 57106 | Spain | Europe | 2016 | 26 | male | uncomplicated gonorrhoea | rectal swab |  |  |
| 57107 | Spain | Europe | 2016 | 28 | male | uncomplicated gonorrhoea | rectal swab |  |  |
| 57108 | Spain | Europe | 2016 | 30 | male | uncomplicated gonorrhoea | rectal swab |  |  |
| 57109 | Spain | Europe | 2016 | 49 | male | uncomplicated gonorrhoea | NA |  |  |
| 57110 | Spain | Europe | 2016 | 24 | male | uncomplicated gonorrhoea | NA |  |  |
| 57111 | Spain | Europe | 2016 | 46 | male | uncomplicated gonorrhoea | NA |  |  |
| 57112 | Spain | Europe | 2016 | 44 | male | uncomplicated gonorrhoea | NA |  |  |
| 57113 | Spain | Europe | 2016 | 39 | male | uncomplicated gonorrhoea | NA |  |  |
| 57114 | Spain | Europe | 2016 | 40 | male | uncomplicated gonorrhoea | NA |  |  |
| 57115 | Spain | Europe | 2016 | 26 | male | uncomplicated gonorrhoea | NA |  |  |
| 57116 | Spain | Europe | 2016 | 33 | male | uncomplicated gonorrhoea | NA |  |  |
| 57117 | Spain | Europe | 2016 | 26 | male | uncomplicated gonorrhoea | NA |  |  |
| 57118 | Spain | Europe | 2016 | 31 | male | uncomplicated gonorrhoea | NA |  |  |
| 57119 | Spain | Europe | 2016 | 34 | male | uncomplicated gonorrhoea | NA |  |  |
| 57120 | Spain | Europe | 2016 | 24 | male | uncomplicated gonorrhoea | NA |  |  |
| 57121 | Spain | Europe | 2016 | 36 | male | uncomplicated gonorrhoea | NA |  |  |
| 57122 | Spain | Europe | 2016 | 22 | male | uncomplicated gonorrhoea | NA |  |  |
| 57123 | Spain | Europe | 2016 | 24 | male | uncomplicated gonorrhoea | NA |  |  |
| 61044 | Norway | Europe | 2017 | 29 | male | NA | urethral swab |  |  |
| 61050 | Norway | Europe | 2017 | 31 | male | NA | urethral swab |  |  |
| 61055 | Norway | Europe | 2017 | 26 | male | NA | urethral swab |  |  |
| 61061 | Norway | Europe | 2017 | 32 | male | NA | urethral swab |  |  |
| 61068 | Norway | Europe | 2017 | 29 | male | NA | urethral swab |  |  |
| 76247 | The Netherlands | Europe | 2013 | 33 | male | NA | urethral swab |  |  |
| 76248 | The Netherlands | Europe | 2013 | 26 | male | NA | rectal swab |  |  |
| 76249 | The Netherlands | Europe | 2013 | 27 | male | NA | rectal swab |  |  |
| 76250 | The Netherlands | Europe | 2013 | 39 | male | NA | rectal swab |  |  |
| 76251 | The Netherlands | Europe | 2013 | 33 | male | NA | rectal swab |  |  |
| 76252 | The Netherlands | Europe | 2013 | 58 | male | NA | urethral swab |  |  |
| 76253 | The Netherlands | Europe | 2013 | 50 | male | NA | rectal swab |  |  |
| 76254 | The Netherlands | Europe | 2013 | 27 | male | NA | rectal swab |  |  |
| 76255 | The Netherlands | Europe | 2013 | 50 | male | NA | urethral swab |  |  |
| 76256 | The Netherlands | Europe | 2013 | 23 | male | NA | urethral swab |  |  |
| 76257 | The Netherlands | Europe | 2013 | 37 | male | NA | rectal swab |  |  |
| 76259 | The Netherlands | Europe | 2013 | 25 | male | NA | rectal swab |  |  |
| 76260 | The Netherlands | Europe | 2013 | 33 | male | NA | rectal swab |  |  |
| 76261 | The Netherlands | Europe | 2013 | 22 | male | NA | rectal swab |  |  |
| 76262 | The Netherlands | Europe | 2013 | 36 | male | NA | rectal swab |  |  |
| 76263 | The Netherlands | Europe | 2013 | 49 | male | NA | throat swab |  |  |
| 76264 | The Netherlands | Europe | 2013 | 32 | male | NA | rectal swab |  |  |
| 76265 | The Netherlands | Europe | 2013 | 50 | male | NA | rectal swab |  |  |
| 76266 | The Netherlands | Europe | 2013 | 56 | male | NA | rectal swab |  |  |
| 76267 | The Netherlands | Europe | 2013 | 31 | male | NA | throat swab |  |  |
| 76270 | The Netherlands | Europe | 2014 | 29 | male | NA | urethral swab |  |  |
| 76271 | The Netherlands | Europe | 2014 | 21 | male | NA | rectal swab |  |  |
| 76272 | The Netherlands | Europe | 2014 | 38 | male | NA | rectal swab |  |  |
| 76273 | The Netherlands | Europe | 2014 | 24 | male | NA | urethral swab |  |  |
| 76274 | The Netherlands | Europe | 2014 | 21 | male | NA | throat swab |  |  |
| 76275 | The Netherlands | Europe | 2014 | 28 | male | NA | urethral swab |  |  |
| 76276 | The Netherlands | Europe | 2014 | 28 | male | NA | rectal swab |  |  |
| 76277 | The Netherlands | Europe | 2014 | 25 | male | NA | throat swab |  |  |
| 76278 | The Netherlands | Europe | 2014 | 47 | male | NA | rectal swab |  |  |
| 76279 | The Netherlands | Europe | 2014 | 38 | male | NA | rectal swab |  |  |
| 76280 | The Netherlands | Europe | 2015 | 32 | male | NA | throat swab |  |  |
| 76281 | The Netherlands | Europe | 2015 | 37 | male | NA | rectal swab |  |  |
| 76282 | The Netherlands | Europe | 2015 | 29 | male | NA | rectal swab |  |  |
| 76283 | The Netherlands | Europe | 2015 | 21 | male | NA | rectal swab |  |  |
| 76284 | The Netherlands | Europe | 2015 | 37 | male | NA | rectal swab |  |  |
| 76289 | The Netherlands | Europe | 2013 | 31 | male | NA | rectal swab |  |  |
| 76290 | The Netherlands | Europe | 2013 | 50 | male | NA | urethral swab |  |  |
| 76291 | The Netherlands | Europe | 2014 | 50 | male | NA | rectal swab |  |  |
| 76297 | Norway | Europe | 2017 | 24 | male | NA | urethral swab |  |  |
| 76312 | Norway | Europe | 2017 | 55 | male | NA | urethral swab |  |  |
| 76315 | Norway | Europe | 2017 | 33 | male | NA | urethral swab |  |  |
| 76316 | Norway | Europe | 2017 | 46 | male | NA | urethral swab |  |  |
| 76321 | Norway | Europe | 2017 | 28 | male | NA | urethral swab |  |  |
| 76322 | Norway | Europe | 2017 | 34 | male | NA | urethral swab |  |  |
| 76328 | Norway | Europe | 2017 | 48 | male | NA | urethral swab |  |  |
| 76330 | Norway | Europe | 2017 | 30 | male | NA | urethral swab |  |  |
| 76337 | Norway | Europe | 2017 | 25 | male | NA | urethral swab |  |  |
| 76340 | Norway | Europe | 2017 | 37 | male | NA | urethral swab |  |  |
| 76344 | Norway | Europe | 2017 | 26 | male | NA | rectal swab |  |  |
| 76345 | Norway | Europe | 2017 | 25 | male | NA | urethral swab |  |  |
| 76346 | Norway | Europe | 2017 | 22 | male | NA | urethral swab |  |  |
| 76353 | Norway | Europe | 2017 | 23 | male | NA | urethral swab |  |  |
| 76355 | Norway | Europe | 2017 | 57 | male | NA | urethral swab |  |  |
| 76358 | Norway | Europe | 2017 | 28 | male | NA | throat swab |  |  |
| 76369 | Norway | Europe | 2017 | 25 | male | NA | urethral swab |  |  |
| 76370 | Norway | Europe | 2017 | 70 | male | NA | throat swab |  |  |
| 76372 | Norway | Europe | 2017 | 30 | male | NA | urethral swab |  |  |
| 76375 | Norway | Europe | 2017 | 47 | male | NA | urethral swab |  |  |
| 76386 | Norway | Europe | 2017 | 24 | male | NA | rectal swab |  |  |
| 76389 | Norway | Europe | 2017 | 54 | male | NA | urethral swab |  |  |
| 76390 | Norway | Europe | 2017 | 28 | male | NA | urethral swab |  |  |
| 76391 | Norway | Europe | 2017 | 52 | male | NA | urethral swab |  |  |
| 76394 | Norway | Europe | 2017 | 31 | male | NA | urethral swab |  |  |
| 76411 | Norway | Europe | 2017 | 40 | male | NA | urethral swab |  |  |
| 76413 | Norway | Europe | 2017 | 26 | female | NA | urethral swab |  |  |
| 76416 | Norway | Europe | 2017 | 38 | male | NA | rectal swab |  |  |
| 76417 | Norway | Europe | 2017 | 36 | male | NA | urethral swab |  |  |
| 76426 | Norway | Europe | 2017 | 29 | male | NA | urethral swab |  |  |
| 76432 | Norway | Europe | 2017 | 42 | male | NA | throat swab |  |  |
| 76433 | Norway | Europe | 2017 | 61 | male | NA | urethral swab |  |  |
| 76435 | Norway | Europe | 2017 | 39 | male | NA | urethral swab |  |  |
| 76443 | Norway | Europe | 2017 | 35 | female | NA | rectal swab |  |  |
| 76445 | Norway | Europe | 2017 | 42 | male | NA | urethral swab |  |  |
| 76452 | Norway | Europe | 2017 | 41 | male | NA | urethral swab |  |  |
| 76461 | Norway | Europe | 2017 | 22 | male | NA | urethral swab |  |  |
| 76462 | Norway | Europe | 2017 | 46 | male | NA | urethral swab |  |  |
| 76464 | Norway | Europe | 2017 | 30 | male | NA | urethral swab |  |  |
| 76476 | Norway | Europe | 2017 | 38 | male | NA | throat swab |  |  |
| 76478 | Norway | Europe | 2017 | 36 | male | NA | urethral swab |  |  |
| 76487 | Norway | Europe | 2017 | 51 | male | NA | rectal swab |  |  |
| 76489 | Norway | Europe | 2017 | 59 | male | NA | rectal swab |  |  |
| 76494 | Norway | Europe | 2017 | 30 | male | NA | urethral swab |  |  |
| 76498 | Norway | Europe | 2017 | 29 | male | NA | rectal swab |  |  |
| 76499 | Norway | Europe | 2017 | 43 | male | NA | throat swab |  |  |
| 76500 | Norway | Europe | 2017 | 52 | male | NA | rectal swab |  |  |
| 76505 | Norway | Europe | 2017 | 23 | male | NA | urethral swab |  |  |
| 76506 | Norway | Europe | 2017 | 27 | male | NA | urethral swab |  |  |
| 76511 | Norway | Europe | 2017 | 53 | male | NA | urethral swab |  |  |
| 76513 | Norway | Europe | 2017 | 64 | male | NA | urethral swab |  |  |
| 76519 | Norway | Europe | 2017 | 26 | male | NA | urethral swab |  |  |
| 77394 | Norway | Europe | 2017 | 33 | male | NA | urethral swab |  |  |
| 77395 | Norway | Europe | 2017 | 44 | male | NA | urethral swab |  |  |
| 77396 | Norway | Europe | 2017 | 56 | male | NA | urethral swab |  |  |
| 77400 | Norway | Europe | 2017 | 72 | male | NA | urethral swab |  |  |
| 77404 | Norway | Europe | 2017 | 48 | male | NA | rectal swab |  |  |
| 77407 | Norway | Europe | 2017 | 22 | male | NA | urethral swab |  |  |
| 89175 | Norway | Europe | 2017 | 52 | male | NA | urethral swab |  |  |
| 108877 | Portugal | Europe | 2013 | 42 | male | uncomplicated gonorrhoea | urethral swab |  |  |
| 108878 | Portugal | Europe | 2013 | 43 | male | uncomplicated gonorrhoea | urethral swab |  |  |
| 108879 | Portugal | Europe | 2013 | 61 | male | uncomplicated gonorrhoea | urethral swab |  |  |
| 108881 | Portugal | Europe | 2014 | 46 | male | disseminated gonococcal infection | blood |  |  |
| 108887 | Portugal | Europe | 2014 | 24 | male | uncomplicated gonorrhoea | throat swab |  |  |
| 108891 | Portugal | Europe | 2014 | 35 | male | uncomplicated gonorrhoea | urethral swab |  |  |
| 108893 | Portugal | Europe | 2014 | 46 | male | uncomplicated gonorrhoea | urethral swab |  |  |
| 108895 | Portugal | Europe | 2014 | 25 | male | uncomplicated gonorrhoea | urethral swab |  |  |
| 108897 | Portugal | Europe | 2014 | 46 | female | uncomplicated gonorrhoea | female reproductive tract |  |  |
| 108899 | Portugal | Europe | 2014 | 35 | male | uncomplicated gonorrhoea | throat swab |  |  |
| 108902 | Portugal | Europe | 2014 | 39 | male | uncomplicated gonorrhoea | urethral swab |  |  |
| 108904 | Portugal | Europe | 2014 | 24 | male | uncomplicated gonorrhoea | urethral swab |  |  |
| 108905 | Portugal | Europe | 2014 | 31 | male | uncomplicated gonorrhoea | urethral swab |  |  |
| 108906 | Portugal | Europe | 2014 | 27 | male | uncomplicated gonorrhoea | urethral swab |  |  |
| 108909 | Portugal | Europe | 2014 | 39 | male | uncomplicated gonorrhoea | urethral swab |  |  |
| 108912 | Portugal | Europe | 2015 | 28 | male | uncomplicated gonorrhoea | urethral swab |  |  |
| 108914 | Portugal | Europe | 2015 | 38 | female | uncomplicated gonorrhoea | female reproductive tract |  |  |
| 108915 | Portugal | Europe | 2015 | 24 | male | uncomplicated gonorrhoea | urethral swab |  |  |
| 108916 | Portugal | Europe | 2015 | 42 | male | uncomplicated gonorrhoea | urethral swab |  |  |
| 108921 | Portugal | Europe | 2015 | 30 | male | uncomplicated gonorrhoea | urethral swab |  |  |
| 108922 | Portugal | Europe | 2015 | 43 | male | uncomplicated gonorrhoea | urethral swab |  |  |
| 108923 | Portugal | Europe | 2015 | 35 | male | uncomplicated gonorrhoea | urethral swab |  |  |
| 108925 | Portugal | Europe | 2015 | 26 | male | uncomplicated gonorrhoea | urethral swab |  |  |
| 108926 | Portugal | Europe | 2015 | 24 | male | uncomplicated gonorrhoea | throat swab |  |  |
| 108927 | Portugal | Europe | 2015 | 41 | female | uncomplicated gonorrhoea | female reproductive tract |  |  |
| 108928 | Portugal | Europe | 2015 | 49 | male | uncomplicated gonorrhoea | urethral swab |  |  |
| 108929 | Portugal | Europe | 2015 | 31 | male | uncomplicated gonorrhoea | urethral swab |  |  |
| 108930 | Portugal | Europe | 2015 | 39 | male | uncomplicated gonorrhoea | urethral swab |  |  |
| 108931 | Portugal | Europe | 2015 | 26 | male | uncomplicated gonorrhoea | urethral swab |  |  |
| 108932 | Portugal | Europe | 2015 | 52 | female | uncomplicated gonorrhoea | female reproductive tract |  |  |
| 108933 | Portugal | Europe | 2015 | 43 | female | uncomplicated gonorrhoea | female reproductive tract |  |  |
| 108934 | Portugal | Europe | 2015 | 36 | male | uncomplicated gonorrhoea | urethral swab |  |  |
| 108937 | Portugal | Europe | 2015 | 46 | male | uncomplicated gonorrhoea | throat swab |  |  |
| 108940 | Portugal | Europe | 2015 | 29 | male | uncomplicated gonorrhoea | urethral swab |  |  |
| 108941 | Portugal | Europe | 2015 | 32 | male | uncomplicated gonorrhoea | urethral swab |  |  |
| 108942 | Portugal | Europe | 2015 | 33 | male | uncomplicated gonorrhoea | urethral swab |  |  |
| 108945 | Portugal | Europe | 2016 | 23 | male | uncomplicated gonorrhoea | rectal swab |  |  |
| 108946 | Portugal | Europe | 2016 | 30 | male | uncomplicated gonorrhoea | urethral swab |  |  |
| 108948 | Portugal | Europe | 2016 | 23 | male | uncomplicated gonorrhoea | urethral swab |  |  |
| 108949 | Portugal | Europe | 2016 | 21 | male | uncomplicated gonorrhoea | throat swab |  |  |
| 108950 | Portugal | Europe | 2016 | 25 | male | uncomplicated gonorrhoea | rectal swab |  |  |
| 108951 | Portugal | Europe | 2016 | 40 | male | uncomplicated gonorrhoea | urethral swab |  |  |
| 108952 | Portugal | Europe | 2016 | 28 | male | uncomplicated gonorrhoea | urethral swab |  |  |
| 108953 | Portugal | Europe | 2016 | 35 | male | uncomplicated gonorrhoea | rectal swab |  |  |
| 108954 | Portugal | Europe | 2016 | 37 | male | uncomplicated gonorrhoea | urethral swab |  |  |
| 108957 | Portugal | Europe | 2016 | 25 | male | uncomplicated gonorrhoea | rectal swab |  |  |
| 108958 | Portugal | Europe | 2016 | 29 | male | uncomplicated gonorrhoea | rectal swab |  |  |
| 108960 | Portugal | Europe | 2016 | 48 | male | uncomplicated gonorrhoea | urethral swab |  |  |
| 108961 | Portugal | Europe | 2016 | 26 | male | uncomplicated gonorrhoea | urethral swab |  |  |
| 108966 | Portugal | Europe | 2016 | 32 | male | uncomplicated gonorrhoea | urethral swab |  |  |
| 108967 | Portugal | Europe | 2016 | 22 | male | uncomplicated gonorrhoea | rectal swab |  |  |
| 108969 | Portugal | Europe | 2016 | 38 | male | uncomplicated gonorrhoea | rectal swab |  |  |
| 108971 | Portugal | Europe | 2016 | 21 | male | uncomplicated gonorrhoea | rectal swab |  |  |
| 108972 | Portugal | Europe | 2016 | 30 | male | uncomplicated gonorrhoea | throat swab |  |  |
| 108974 | Portugal | Europe | 2017 | 36 | female | uncomplicated gonorrhoea | female reproductive tract |  |  |
| 108975 | Portugal | Europe | 2017 | 63 | female | uncomplicated gonorrhoea | female reproductive tract |  |  |
| 108976 | Portugal | Europe | 2017 | 35 | female | uncomplicated gonorrhoea | female reproductive tract |  |  |
| 108977 | Portugal | Europe | 2017 | 32 | female | uncomplicated gonorrhoea | female reproductive tract |  |  |
| 108978 | Portugal | Europe | 2017 | 41 | male | uncomplicated gonorrhoea | urethral swab |  |  |
| 108979 | Portugal | Europe | 2017 | 23 | female | uncomplicated gonorrhoea | female reproductive tract |  |  |
| 108981 | Portugal | Europe | 2017 | 52 | female | uncomplicated gonorrhoea | female reproductive tract |  |  |
| 108982 | Portugal | Europe | 2017 | 40 | female | uncomplicated gonorrhoea | female reproductive tract |  |  |
| 108983 | Portugal | Europe | 2017 | 35 | female | uncomplicated gonorrhoea | female reproductive tract |  |  |
| 108984 | Portugal | Europe | 2017 | 35 | male | uncomplicated gonorrhoea | urethral swab |  |  |
| 108985 | Portugal | Europe | 2017 | 34 | female | uncomplicated gonorrhoea | female reproductive tract |  |  |
| 108986 | Portugal | Europe | 2017 | 42 | male | uncomplicated gonorrhoea | urethral swab |  |  |
| 108987 | Portugal | Europe | 2017 | 60 | male | uncomplicated gonorrhoea | urethral swab |  |  |
| 108988 | Portugal | Europe | 2017 | 38 | male | uncomplicated gonorrhoea | urethral swab |  |  |
| 108989 | Portugal | Europe | 2017 | 35 | female | uncomplicated gonorrhoea | female reproductive tract |  |  |
| 108991 | Portugal | Europe | 2017 | 29 | male | uncomplicated gonorrhoea | throat swab |  |  |
| 108992 | Portugal | Europe | 2017 | 22 | male | uncomplicated gonorrhoea | urethral swab |  |  |
| 108993 | Portugal | Europe | 2017 | 30 | male | uncomplicated gonorrhoea | urethral swab |  |  |
| 108994 | Portugal | Europe | 2017 | 31 | male | uncomplicated gonorrhoea | urethral swab |  |  |
| 108995 | Portugal | Europe | 2017 | 23 | male | uncomplicated gonorrhoea | urethral swab |  |  |
| 108996 | Portugal | Europe | 2017 | 33 | male | uncomplicated gonorrhoea | urethral swab |  |  |
| 108997 | Portugal | Europe | 2017 | 27 | male | uncomplicated gonorrhoea | urethral swab |  |  |
| 108999 | Portugal | Europe | 2017 | 44 | male | uncomplicated gonorrhoea | urethral swab |  |  |
| 109000 | Portugal | Europe | 2017 | 24 | male | uncomplicated gonorrhoea | urethral swab |  |  |
| 109005 | Portugal | Europe | 2017 | 45 | male | uncomplicated gonorrhoea | urethral swab |  |  |
| 109007 | Portugal | Europe | 2017 | 46 | male | uncomplicated gonorrhoea | urethral swab |  |  |
| 109009 | Portugal | Europe | 2017 | 25 | male | uncomplicated gonorrhoea | urethral swab |  |  |
| 109011 | Portugal | Europe | 2017 | 49 | male | uncomplicated gonorrhoea | urethral swab |  |  |
| 109012 | Portugal | Europe | 2017 | 37 | male | uncomplicated gonorrhoea | urethral swab |  |  |
| 109014 | Portugal | Europe | 2017 | 22 | male | uncomplicated gonorrhoea | urethral swab |  |  |
| 109015 | Portugal | Europe | 2017 | 40 | female | uncomplicated gonorrhoea | female reproductive tract |  |  |
| 109017 | Portugal | Europe | 2017 | 42 | male | uncomplicated gonorrhoea | urethral swab |  |  |
| 109018 | Portugal | Europe | 2017 | 30 | female | uncomplicated gonorrhoea | female reproductive tract |  |  |
| 109019 | Portugal | Europe | 2017 | 56 | female | uncomplicated gonorrhoea | female reproductive tract |  |  |
| 109023 | Portugal | Europe | 2017 | 29 | male | uncomplicated gonorrhoea | urethral swab |  |  |
| 109024 | Portugal | Europe | 2017 | 30 | male | uncomplicated gonorrhoea | rectal swab |  |  |
| 109027 | Portugal | Europe | 2017 | 35 | male | uncomplicated gonorrhoea | urethral swab |  |  |
| 109028 | Portugal | Europe | 2017 | 59 | female | uncomplicated gonorrhoea | female reproductive tract |  |  |
| 109029 | Portugal | Europe | 2017 | 28 | male | uncomplicated gonorrhoea | urethral swab |  |  |
| 109030 | Portugal | Europe | 2017 | 21 | male | uncomplicated gonorrhoea | urethral swab |  |  |
| 109033 | Portugal | Europe | 2017 | 25 | male | uncomplicated gonorrhoea | urethral swab |  |  |
| 109038 | Portugal | Europe | 2017 | 26 | female | uncomplicated gonorrhoea | female reproductive tract |  |  |
| 109039 | Portugal | Europe | 2017 | 27 | female | uncomplicated gonorrhoea | female reproductive tract |  |  |
| 109040 | Portugal | Europe | 2017 | 51 | male | uncomplicated gonorrhoea | urethral swab |  |  |
| 109041 | Portugal | Europe | 2017 | 32 | male | uncomplicated gonorrhoea | urethral swab |  |  |
| 109042 | Portugal | Europe | 2017 | 29 | male | uncomplicated gonorrhoea | rectal swab |  |  |
| 109043 | Portugal | Europe | 2017 | 21 | male | uncomplicated gonorrhoea | urethral swab |  |  |
| 109044 | Portugal | Europe | 2017 | 56 | female | uncomplicated gonorrhoea | female reproductive tract |  |  |
| 109047 | Portugal | Europe | 2017 | 39 | male | uncomplicated gonorrhoea | urethral swab |  |  |
| 109049 | Portugal | Europe | 2017 | 26 | male | uncomplicated gonorrhoea | urethral swab |  |  |
| 109050 | Portugal | Europe | 2017 | 24 | male | uncomplicated gonorrhoea | urethral swab |  |  |
| 109051 | Portugal | Europe | 2017 | 37 | male | uncomplicated gonorrhoea | urethral swab |  |  |
| 109054 | Portugal | Europe | 2017 | 39 | male | uncomplicated gonorrhoea | urethral swab |  |  |
| 109055 | Portugal | Europe | 2017 | 31 | male | uncomplicated gonorrhoea | urethral swab |  |  |
| 109059 | Portugal | Europe | 2017 | 58 | male | uncomplicated gonorrhoea | urethral swab |  |  |
| 109064 | Portugal | Europe | 2017 | 43 | male | uncomplicated gonorrhoea | urethral swab |  |  |
| 109065 | Portugal | Europe | 2017 | 26 | male | uncomplicated gonorrhoea | urethral swab |  |  |
| 109069 | Portugal | Europe | 2017 | 40 | male | uncomplicated gonorrhoea | urethral swab |  |  |
| 109071 | Portugal | Europe | 2017 | 23 | female | uncomplicated gonorrhoea | female reproductive tract |  |  |
| 109072 | Portugal | Europe | 2017 | 28 | male | uncomplicated gonorrhoea | urethral swab |  |  |
| 109073 | Portugal | Europe | 2017 | 28 | male | uncomplicated gonorrhoea | rectal swab |  |  |
| 109074 | Portugal | Europe | 2017 | 26 | male | uncomplicated gonorrhoea | rectal swab |  |  |
| 109076 | Portugal | Europe | 2017 | 22 | male | uncomplicated gonorrhoea | rectal swab |  |  |
| 109077 | Portugal | Europe | 2017 | 26 | male | uncomplicated gonorrhoea | urethral swab |  |  |
| 109080 | Portugal | Europe | 2017 | 29 | male | uncomplicated gonorrhoea | urethral swab |  |  |
| 109081 | Portugal | Europe | 2017 | 36 | male | uncomplicated gonorrhoea | urethral swab |  |  |
| 109084 | Portugal | Europe | 2017 | 23 | female | uncomplicated gonorrhoea | female reproductive tract |  |  |
| 109087 | Portugal | Europe | 2017 | 50 | male | uncomplicated gonorrhoea | urethral swab |  |  |
| 109090 | Portugal | Europe | 2017 | 26 | male | uncomplicated gonorrhoea | urethral swab |  |  |
| 109091 | Portugal | Europe | 2017 | 74 | male | uncomplicated gonorrhoea | urethral swab |  |  |
| 109096 | Portugal | Europe | 2017 | 21 | female | uncomplicated gonorrhoea | female reproductive tract |  |  |
| 115835 | Ireland | Europe | 2018 | NA | male | uncomplicated gonorrhoea | urethral swab |  |  |
| 115836 | Spain | Europe | 2016 | 32 | male | NA | NA |  |  |
| 115837 | Spain | Europe | 2016 | 36 | male | NA | NA |  |  |
| 115838 | Spain | Europe | 2016 | 31 | male | NA | NA |  |  |
| 115839 | Spain | Europe | 2016 | 28 | male | NA | NA |  |  |
| 115840 | Spain | Europe | 2016 | 24 | male | NA | NA |  |  |
| 115841 | Spain | Europe | 2016 | 40 | male | NA | NA |  |  |
| 115842 | Spain | Europe | 2017 | 43 | male | NA | NA |  |  |
| 115843 | Spain | Europe | 2017 | 24 | male | NA | NA |  |  |
| 115844 | Spain | Europe | 2017 | 28 | male | NA | NA |  |  |
| 115845 | Spain | Europe | 2017 | 46 | male | NA | NA |  |  |
| 115846 | Spain | Europe | 2017 | 34 | male | NA | NA |  |  |
| 115847 | Spain | Europe | 2017 | 35 | male | NA | NA |  |  |
| 115848 | Spain | Europe | 2017 | 43 | male | NA | NA |  |  |
| 115849 | Spain | Europe | 2017 | 29 | male | NA | NA |  |  |
| 115850 | Spain | Europe | 2017 | 26 | male | NA | NA |  |  |
| 115866 | Ireland | Europe | 2012 | NA | male | NA | urethral swab |  |  |
| 116257 | Germany | Europe | 2018 | 35 | male | NA | urethral swab |  |  |
| 116258 | Germany | Europe | 2018 | 23 | male | NA | rectal swab |  |  |
| 116259 | Germany | Europe | 2018 | 63 | male | NA | urethral swab |  |  |
| 116260 | Germany | Europe | 2018 | 60 | male | NA | urethral swab |  |  |
| 116263 | Germany | Europe | 2018 | 45 | male | NA | urethral swab |  |  |
| 116264 | Germany | Europe | 2018 | 62 | female | NA | female reproductive tract |  |  |
| 116265 | Germany | Europe | 2018 | 36 | male | NA | urethral swab |  |  |
| 116266 | Germany | Europe | 2018 | 48 | male | NA | urethral swab |  |  |
| 116267 | Germany | Europe | 2018 | 46 | male | NA | urethral swab |  |  |
| 116268 | Germany | Europe | 2018 | 39 | male | NA | urethral swab |  |  |
| 116269 | Germany | Europe | 2018 | 28 | male | NA | urethral swab |  |  |
| 116270 | Germany | Europe | 2018 | 31 | male | NA | urethral swab |  |  |
| 116271 | Germany | Europe | 2018 | 21 | male | NA | urethral swab |  |  |
| 116272 | Germany | Europe | 2018 | 27 | male | NA | urethral swab |  |  |
| 116273 | Germany | Europe | 2018 | 28 | male | NA | urethral swab |  |  |
| 116274 | Germany | Europe | 2018 | 52 | male | NA | urethral swab |  |  |
| 116275 | Germany | Europe | 2018 | 39 | male | NA | urethral swab |  |  |
| 116276 | Germany | Europe | 2018 | 62 | male | NA | urethral swab |  |  |
| 116277 | Germany | Europe | 2018 | 29 | male | NA | urethral swab |  |  |
| 116278 | Germany | Europe | 2018 | 42 | male | NA | urethral swab |  |  |
| 116279 | Germany | Europe | 2018 | 46 | male | NA | urethral swab |  |  |
| 116280 | Germany | Europe | 2018 | 51 | male | NA | urethral swab |  |  |
| 116281 | Germany | Europe | 2018 | 23 | male | NA | urethral swab |  |  |
| 116282 | Germany | Europe | 2018 | 34 | male | NA | urethral swab |  |  |
| 116283 | Germany | Europe | 2018 | 23 | male | NA | urethral swab |  |  |
| 116286 | Germany | Europe | 2018 | 53 | female | NA | female reproductive tract |  |  |
| 116287 | Germany | Europe | 2018 | 34 | male | NA | urethral swab |  |  |
| 116289 | Germany | Europe | 2018 | 59 | male | NA | urethral swab |  |  |
| 116290 | Germany | Europe | 2018 | 20 | male | NA | urethral swab |  |  |
| 116291 | Germany | Europe | 2018 | 39 | male | NA | urethral swab |  |  |
| 116292 | Germany | Europe | 2018 | 27 | male | NA | urethral swab |  |  |
| 116293 | Germany | Europe | 2018 | 36 | male | NA | urethral swab |  |  |
| 116294 | Germany | Europe | 2018 | 50 | male | NA | rectal swab |  |  |
| 116296 | Germany | Europe | 2018 | 43 | male | NA | urethral swab |  |  |
| 116297 | Germany | Europe | 2018 | 32 | male | NA | urethral swab |  |  |
| 116298 | Germany | Europe | 2018 | 27 | male | NA | urethral swab |  |  |
| 116299 | Germany | Europe | 2018 | 46 | male | NA | urethral swab |  |  |
| 116302 | Germany | Europe | 2018 | 27 | male | NA | urethral swab |  |  |
| 116303 | Germany | Europe | 2018 | 28 | male | NA | urethral swab |  |  |
| 116304 | Germany | Europe | 2018 | 38 | male | NA | urethral swab |  |  |
| 116305 | Germany | Europe | 2018 | 32 | male | NA | urethral swab |  |  |
| 116306 | Germany | Europe | 2018 | 32 | male | NA | urethral swab |  |  |
| 116307 | Germany | Europe | 2018 | 26 | male | NA | urethral swab |  |  |
| 116309 | Germany | Europe | 2018 | 28 | male | NA | urethral swab |  |  |
| 116310 | Germany | Europe | 2018 | 42 | male | NA | urethral swab |  |  |
| 116311 | Germany | Europe | 2018 | 25 | male | NA | urethral swab |  |  |
| 116312 | Germany | Europe | 2018 | 45 | male | NA | urethral swab |  |  |
| 116313 | Germany | Europe | 2018 | 22 | male | NA | urethral swab |  |  |
| 116314 | Germany | Europe | 2018 | 33 | male | NA | rectal swab |  |  |
| 116338 | Germany | Europe | 2018 | 25 | female | NA | female reproductive tract |  |  |
| 41377 | Italy | Europe | 2013 | 45 | NA | uncomplicated gonorrhoea | throat swab |  |  |
| 41385 | Italy | Europe | 2014 | 29 | NA | uncomplicated gonorrhoea | throat swab |  |  |
| 41387 | Italy | Europe | 2014 | 31 | male | uncomplicated gonorrhoea | urethral swab |  |  |
| 41395 | Italy | Europe | 2014 | 31 | NA | uncomplicated gonorrhoea | throat swab |  |  |
| 41396 | Italy | Europe | 2014 | 73 | NA | uncomplicated gonorrhoea | throat swab |  |  |
| 41400 | Italy | Europe | 2014 | 45 | NA | disseminated gonococcal infection | blood |  |  |
| 41404 | Italy | Europe | 2014 | 37 | male | uncomplicated gonorrhoea | urethral swab |  |  |
| 41409 | Italy | Europe | 2014 | 31 | NA | uncomplicated gonorrhoea | throat swab |  |  |
| 60478 | Italy | Europe | 2015 | 28 | male | uncomplicated gonorrhoea | urethral swab |  |  |
| 60479 | Italy | Europe | 2016 | 29 | male | uncomplicated gonorrhoea | urethral swab |  |  |
| 60480 | Italy | Europe | 2017 | 34 | male | uncomplicated gonorrhoea | urethral swab |  |  |
| 60481 | Italy | Europe | 2017 | 46 | male | uncomplicated gonorrhoea | urethral swab |  |  |
| 60482 | Italy | Europe | 2017 | 55 | male | uncomplicated gonorrhoea | urethral swab |  |  |
| 60483 | Italy | Europe | 2017 | 40 | male | uncomplicated gonorrhoea | urethral swab |  |  |
| 60484 | Italy | Europe | 2017 | 29 | male | uncomplicated gonorrhoea | urethral swab |  |  |
| 60485 | Italy | Europe | 2017 | 28 | male | uncomplicated gonorrhoea | urethral swab |  |  |
| 60486 | Italy | Europe | 2017 | 31 | male | uncomplicated gonorrhoea | urethral swab |  |  |
| 60487 | Italy | Europe | 2017 | 44 | male | uncomplicated gonorrhoea | urethral swab |  |  |
| 60488 | Italy | Europe | 2017 | 45 | female | pelvic infiammatory disease | female reproductive tract |  |  |
| 60489 | Italy | Europe | 2017 | 26 | male | uncomplicated gonorrhoea | urethral swab |  |  |
| 60490 | Italy | Europe | 2017 | 35 | NA | uncomplicated gonorrhoea | rectal swab |  |  |
| 60491 | Italy | Europe | 2017 | 61 | male | uncomplicated gonorrhoea | urethral swab |  |  |
| 60492 | Italy | Europe | 2017 | 57 | male | uncomplicated gonorrhoea | urethral swab |  |  |
| 60493 | Italy | Europe | 2017 | 20 | male | uncomplicated gonorrhoea | urethral swab |  |  |
| 60494 | Italy | Europe | 2017 | 20 | male | uncomplicated gonorrhoea | urethral swab |  |  |
| 60495 | Italy | Europe | 2017 | 21 | male | uncomplicated gonorrhoea | urethral swab |  |  |
| 60496 | Italy | Europe | 2017 | 37 | male | uncomplicated gonorrhoea | urethral swab |  |  |
| 60497 | Italy | Europe | 2017 | 26 | male | uncomplicated gonorrhoea | urethral swab |  |  |
| 60498 | Italy | Europe | 2017 | 35 | male | uncomplicated gonorrhoea | urethral swab |  |  |
| 60499 | Italy | Europe | 2017 | 22 | female | pelvic infiammatory disease | female reproductive tract |  |  |
| 41378 | Italy | Europe | 2013 | 56 | male | uncomplicated gonorrhoea | urethral swab |  |  |
| 41379 | Italy | Europe | 2013 | 25 | NA | uncomplicated gonorrhoea | rectal swab |  |  |
| 41380 | Italy | Europe | 2013 | 39 | male | uncomplicated gonorrhoea | urethral swab |  |  |
| 41381 | Italy | Europe | 2013 | 36 | male | uncomplicated gonorrhoea | urethral swab |  |  |
| 41383 | Italy | Europe | 2013 | 44 | NA | uncomplicated gonorrhoea | rectal swab |  |  |
| 41384 | Italy | Europe | 2013 | 21 | male | uncomplicated gonorrhoea | urethral swab |  |  |
| 41386 | Italy | Europe | 2014 | 29 | male | uncomplicated gonorrhoea | urethral swab |  |  |
| 41388 | Italy | Europe | 2014 | 31 | NA | uncomplicated gonorrhoea | rectal swab |  |  |
| 41389 | Italy | Europe | 2014 | 31 | male | uncomplicated gonorrhoea | urethral swab |  |  |
| 41390 | Italy | Europe | 2014 | 25 | NA | uncomplicated gonorrhoea | rectal swab |  |  |
| 41391 | Italy | Europe | 2014 | 43 | male | uncomplicated gonorrhoea | urethral swab |  |  |
| 41392 | Italy | Europe | 2014 | 21 | NA | pelvic infiammatory disease | rectal swab |  |  |
| 41393 | Italy | Europe | 2014 | 23 | NA | uncomplicated gonorrhoea | rectal swab |  |  |
| 41394 | Italy | Europe | 2014 | 33 | NA | uncomplicated gonorrhoea | rectal swab |  |  |
| 41397 | Italy | Europe | 2014 | 25 | male | uncomplicated gonorrhoea | urethral swab |  |  |
| 41398 | Italy | Europe | 2014 | 21 | male | uncomplicated gonorrhoea | urethral swab |  |  |
| 41399 | Italy | Europe | 2014 | 39 | male | uncomplicated gonorrhoea | urethral swab |  |  |
| 41401 | Italy | Europe | 2014 | 29 | NA | uncomplicated gonorrhoea | rectal swab |  |  |
| 41402 | Italy | Europe | 2014 | 38 | NA | uncomplicated gonorrhoea | rectal swab |  |  |
| 41403 | Italy | Europe | 2014 | 24 | male | uncomplicated gonorrhoea | urethral swab |  |  |
| 41405 | Italy | Europe | 2014 | 37 | male | uncomplicated gonorrhoea | urethral swab |  |  |
| 41406 | Italy | Europe | 2014 | 37 | NA | uncomplicated gonorrhoea | rectal swab |  |  |
| 41407 | Italy | Europe | 2014 | 30 | male | uncomplicated gonorrhoea | urethral swab |  |  |
| 41408 | Italy | Europe | 2014 | 30 | male | uncomplicated gonorrhoea | urethral swab |  |  |
| 41410 | Italy | Europe | 2014 | 35 | male | uncomplicated gonorrhoea | urethral swab |  |  |
