# Supplementary material for: Seven Years of Culture Collection of Neisseria gonorrhoeae: Antimicrobial Resistance and Molecular Epidemiology
Source: Microb Drug Resist. 2023 Mar 16;29(3):85–95. doi: 10.1089/mdr.2021.0483 (PMC10024589; doi:10.1089/mdr.2021.0483)
Supplement: Supplemental data [file Supp_FigS1.docx]

# Figure Legends

# Fig. S1. Box and whiskers plots of the minimum inhibitory concentrations (MICs) of ceftriaxone (A),

# spectinomycin (B) and gentamicin (C) examined for all viable isolates from 2013 to 2019. The

# horizontal dotted lines indicate the European Committee on Antimicrobial Susceptibility Testing

(EUCAST) resistant breakpoint (>0.125mg/L for ceftriaxone and >64mg/L for spectinomycin).

One-way ANOVA was used for the analysis.
