# Supplementary material for: Seven Years of Culture Collection of Neisseria gonorrhoeae: Antimicrobial Resistance and Molecular Epidemiology
Source: Microb Drug Resist. 2023 Mar 16;29(3):85–95. doi: 10.1089/mdr.2021.0483 (PMC10024589; doi:10.1089/mdr.2021.0483)

**Fig. S2** Genomic comparison of representative genomes. Genetic relatedness of a subsample of Italian gonococci (n=55) with a representative panel of European gonococci (n=521) was evaluated.


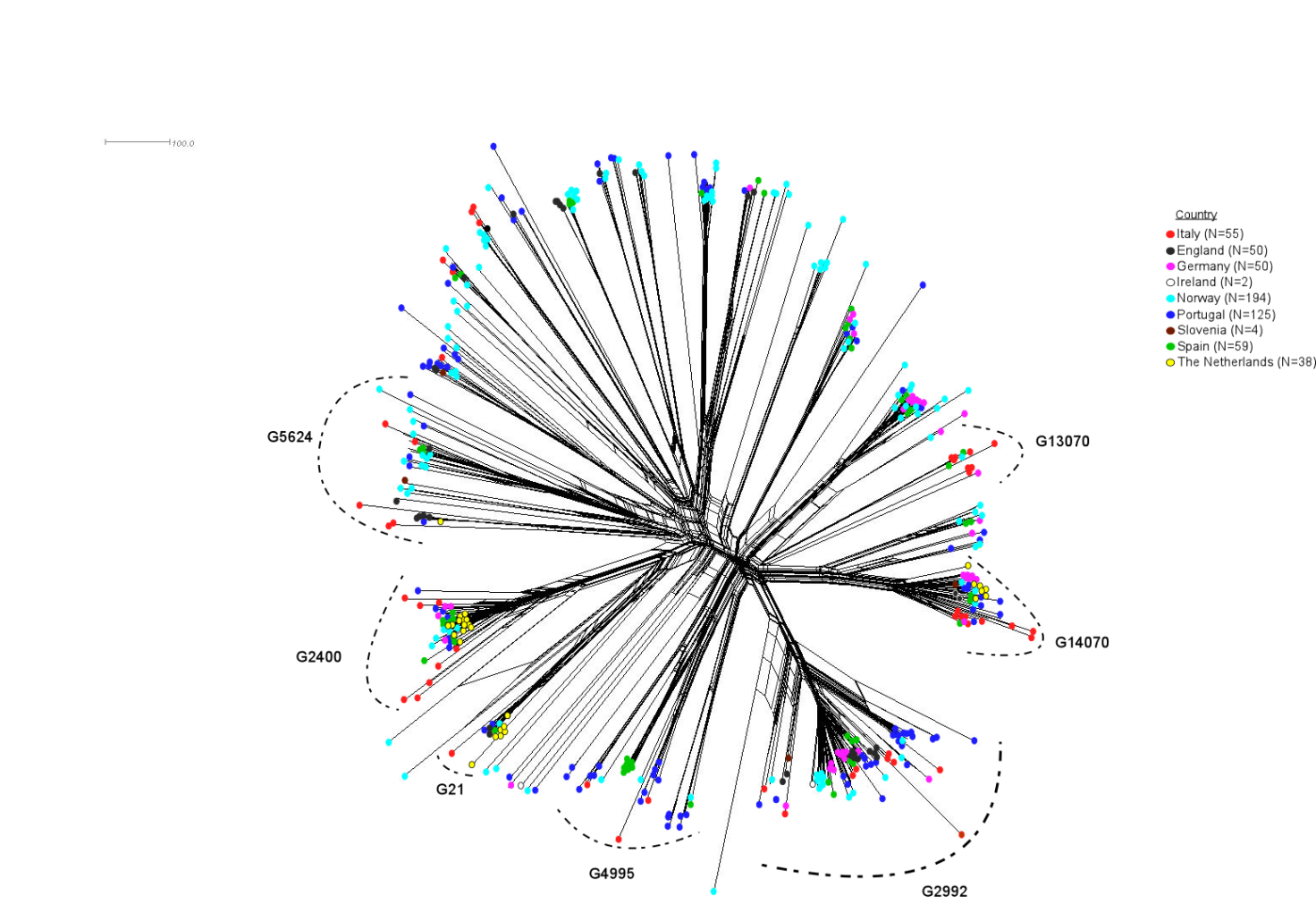

Supplement: Supplemental data [file Supp_FigS2.docx]
